# Molecular Design of Thermoresponsive Poly(oligoethylene glycol)vinyl Esters for the Generation of Polymer Brush-Based Biointerfaces

**DOI:** 10.1021/acspolymersau.6c00053

**Published:** 2026-05-08

**Authors:** Francesco M. Ara, Rachele Foladore, Alessandro Magro, Nicola Scapin, Daniele Rizzi, Alessandro Bertolo, Francesca Lorandi, Edmondo M. Benetti

**Affiliations:** † Laboratory for Macromolecular and Organic Chemistry (MOC), Department of Chemical Sciences, 9308 University of Padova, via Marzolo 1, 35131 Padova, Italy

**Keywords:** poly(vinyl ester)s, controlled radical polymerization, polymer brushes, thermoresponsive polymers, poly(ethylene glycol)

## Abstract

Vinyl polymers bearing oligoethylene glycol (OEG) side chains have emerged as starting materials for the generation of biointerfaces and thermoresponsive systems. Here, we report the molecular design of thermoresponsive poly­(oligoethylene glycol)­vinyl esters (POEGVEs) and evaluate their behavior in solution as well as their performance as surface-grafted polymer brushes. POEGVEs bearing one and two ethylene glycol units were synthesized via RAFT/MADIX polymerization and directly compared to the corresponding polyacrylate analogues. Despite their closely related compositions, POEGVEs exhibited markedly higher LCST values in water, indicating stronger polymer–water interactions associated with the higher polarity of the vinyl ester backbone. When assembled as polymer brush layers on model Au surfaces, POEGVEs displayed enhanced hydration, higher swelling ratios, and lower water contact angles relative to those of the corresponding poly­(oligoethylene glycol)­acrylate (POEGA) brushes. This increased hydration translated into significantly improved resistance to nonspecific protein adsorption from full human serum (FHS). Collectively, these findings identify OEG-functional poly­(vinyl ester)­s as a promising and previously overlooked platform for the design of thermoresponsive and antifouling polymer biointerfaces.

## Graphical abstract

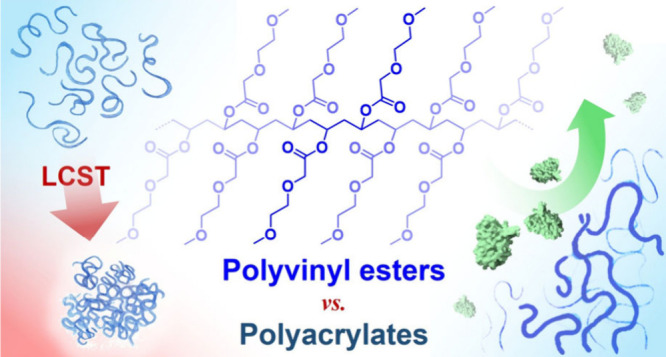

## Introduction

Polyvinyls bearing oligoethylene glycol (OEG) side chains have emerged as highly versatile materials finding a variety of applications in biotechnology, while serving as attractive alternatives to linear polyethylene glycols (PEGs). Among these, poly­(oligoethylene glycol) acrylates (POEGAs) and the corresponding methacrylates (POEGMAs) are the most extensively investigated.^1–9^ The tunable hydrophilic–hydrophobic balance of POEG­(M)­As gives rise to thermoresponsive behavior in aqueous media, while the pronounced hydration associated with the OEG side chains confers intrinsic bioinertness.^1–3^ These characteristics have enabled their widespread use in the design and functionalization of advanced biomaterials, as well as in the fabrication of biosensors and protein-repellent surfaces.^3,8,10^

The lower critical solution temperature (LCST) of POEGAs and POEGMAs can be systematically adjusted by copolymerizing (meth)­acrylate precursors bearing OEG side chains of varying lengths and compositions.^11,12^ This high degree of synthetic modularity has established POEG­(M)­As as model polymers for elucidating structure–thermoresponsive property relationships.^1,13,14^

Besides their relevance in the design of smart materials, POEG­(M)­As exhibit exceptional interfacial properties when immobilized on solid substrates as surface-grafted polymer brushes. Densely grafted POEG­(M)­A brushes form highly hydrated layers that effectively suppress nonspecific protein adsorption and cell adhesion,^8,15–18^ thus enabling their application as bioinert coatings in biomedical and sensing applications.^19–22^ Notably, through the precise adjustment of the OEG side chain length, POEG­(M)­A assemblies show a significant suppression of anti-PEG antibody recognition, providing polymeric platforms that retain the advantageous properties of PEG while mitigating concerns associated with its immunogenicity. This has facilitated the application of POEG­(M)As for the formulation of bioconjugates and the fabrication of therapeutic nanoparticle systems.^9,22–24^

Despite their success, the overwhelming majority of OEG-containing polyvinyl systems rely on poly­(meth)­acrylate backbones. In contrast, poly­(vinyl ester)­s (PVEs)—although industrially established and exemplified by poly­(vinyl acetate) (PVAc)—have been investigated to a far lesser extent. The little attention paid to developing functional, OEG-bearing polymers with vinyl ester backbone is somewhat surprising given that PVEs are known for their degradability under environmental conditions^26,27^ and—in the case of PVAc and related derivatives—established regulatory acceptance in pharmaceutical and biomedical applications.^28^

The intrinsic degradability and well-documented safety profile of vinyl ester-based polymers may offer distinct advantages for transient, resorbable, or biointegrated systems, particularly where the long-term persistence of (meth)­acrylate backbones could be undesirable and could lead to accumulation in physiological environments. Inspired by the opportunity to explore the properties of poly­(oligoethylene glycol) vinyl esters (POEGVEs) and their potential for generating bioinert polymer interfaces, we focused on two representative members of this family bearing side chains with one or two OEG repeating units and comparatively analyzed their thermoresponsive and interfacial properties alongside those of the corresponding polyacrylate analogues.

We specifically focused on POEGVEs bearing short OEG side chains, as this structural feature was previously identified as critical for preventing the formation of epitopes recognized by anti-PEG antibodies (APA), which typically emerge for side chains comprising three or more OEG units.^14,29^ Moreover, relatively short OEG substituents have been reported to enable LCST transitions within a practically accessible range, *i.e.*, between ambient temperature and below 100 °C.^30–32^

A comprehensive analysis of their thermoresponsive behavior in solution as well as their interfacial properties when assembled as polymer brush layers on model substrates revealed that POEGVEs exhibit a more pronounced tendency toward hydration compared to their POEGA counterparts. This enhanced hydration manifested in higher LCST values, increased wettability and swelling of the brush layers, and significantly improved resistance to nonspecific protein adsorption when evaluated against full human serum (FHS).

Collectively, the reported results highlight how POEGVEs represent promising alternatives to the established POEG­(M)­A platform. By combining thermoresponsive behavior and pronounced hydration with a vinyl ester backbone that may offer distinct degradability and regulatory advantages, they represent a conceptually new class of polyvinyls for the design of biointerfaces and the functionalization of biomaterials.

## Experimental Section

### Materials

All reagents, materials, and solvents employed in the syntheses were purchased from commercial suppliers and used as received, without further purification, except for AIBN, which was recrystallized in MeOH.

The following were used: 2-(2-methoxyethoxy)­acetic acid (technical grade, Sigma-Aldrich); KOH (>99%, Merck); Pd­(OAc)_2_ (>99%, Merck); VAc (>99% stab., Merck); [2-(2-methoxyethoxy)­ethoxy]­acetic acid (>95%, Merck); K_2_CO_3_ (≥99%, Sigma-Aldrich); acrylic acid (99% stab. with MEHQ 200 ppm, Sigma-Aldrich); 2-methoxyethoxymethyl chloride (>95%, TCI); LiCl (≥99.0%, Fluka); Na_2_SO_4_ anhydrous (>99%, Merck); 2-(2-methoxyethoxy)­ethyl alcohol (98% stab. with BHT 50–150 mg/kg, Alfa Aesar); p-TsOH (>98%, TCI); BHT (99%, ABCR); hydrogen peroxide (>99%, Merck); NaCl (≥99.0%, Sigma-Aldrich); methyl 2-bromopropanoate (>95%, Merck); potassium *O*-ethyl dithiocarbonate (>95%, TCI); dodecanthiol (≥98, Sigma-Aldrich); TEA (>99%, Merck); CS_2_ (99.9%, Thermo Scientific); HCl (37% in H_2_O, Sigma-Aldrich); AIBN (>99%, Merck); BuNH_2_ (99+%, Acros Organic). All solvents were purchased from Merck or Carlo Erba. Deuterated solvents used for the acquisition of ^1^H and ^13^C NMR spectra were purchased from commercial suppliers (Sigma-Aldrich, Eurisotop) and featured a minimum isotopic purity of 99.8%.

TLC analyses were performed on silica gel 60 F254 aluminum sheets (Merck). Kieselgel 60 M silica (0.063–0.40 mm) was used as the stationary phase for column (flash) chromatography. Visualization of the developed chromatograms was carried out by checking UV absorbance at λ = 254 nm as well as by staining with an aqueous potassium permanganate solution.

Celite (545, Sigma-Aldrich) was used for filtration. Ultrapure water was purified with a Millipore Direct-Q5 ultrapure water system or a Milli-Q IQ 7003 purification system.

### Methods

#### Nuclear Magnetic Resonance (NMR) Spectroscopy

^1^H NMR and ^13^C NMR spectra were recorded using a Bruker AVANCE NANOBAY spectrometer at 400 MHz equipped with a Varian 400/54 (9.39 T) magnet and a BBFO probe, a Bruker AVII400 Ultra-Shield spectrometer at 400 MHz equipped with AVANCE III HD (Bruker) console and a BBI z-grad probe; a Bruker Neo 600 MHz spectrometer, equipped with a NEO (Bruker) console nitrogen cooled 5 mm Prodigy CryoProbe, and a Bruker AVANCE 200 operating at 200 MHz, equipped with an Oxford Instruments superconducting magnet (4.7 T). Chemical shifts (δ) are given in ppm relative to the signal of the residual nondeuterated solvent. Peaks are described as singlet (s), doublet (d), triplet (t), quadruplet (q), quintet (p), and multiplet (m).

#### Size-Exclusion Chromatography (SEC)

SEC was performed using a Shimadzu NEXERA system equipped with two PSS Agilent columns and one Phenogel column (SDV, 300 × 8 mm, pore sizes of 500, 1000, and 105 Å), and a refractive index detector. DMF + LiBr 1 g L^–1^ were used as the mobile phase at a flow rate of 1 mL min^–1^ and a temperature of 60 °C. Instrument calibration was performed using PMMA standards (2680–400000 Da) supplied by Polymer Laboratories Ltd.

#### Quartz Crystal Microbalance with Dissipation (QCMD)

The assembly of polymer adsorbates on Au surfaces and interaction with proteins were monitored in situ by QCMD, using an E4 instrument (Q-Sense AB, Göteborg, Sweden) equipped with dedicated Q-Sense AB software. Au-coated crystals (Biolin Scientific) with a fundamental resonance frequency of 4.95 MHz were used as substrates. Before the experiment, the substrates were cleaned in a UV-cleaner (Boekel Industries, Inc.), immersed in a piranha solution (H_2_SO_4_:H_2_O_2_, 3:1) for 30 s, rinsed with ultrapure water and ethanol, and dried under a stream of N_2_. Then, they were exposed to ultrapure water at ambient temperature until a stable baseline was recorded. Later, ultrapure water was replaced with ethanol, followed by the injection of a 1 mg mL^–1^ polymer solution in ethanol until complete adsorption was attained (1–2 h). To eliminate the physiosorbed polymer, washing steps were performed with ethanol and ultrapure water. A second cycle of injections was performed to evaluate the interaction between the polymer layer and 10% full human serum (FHS). Ultrapure water was replaced with PBS (10 mM), followed by lyophilized FHS in PBS. Later, the substrates were rinsed to remove weakly bound proteins.

The values of hydrated thickness (*T*_wet_) of the polymer layer were obtained by applying an extended viscoelastic model, fitting the frequency shifts (Δ*F*) by using three different overtones (fifth, seventh, and ninth) for each sample. Three crystals for each film were used to calculate the mean values of *T*_wet_ and their standard deviations. The input parameters for the fitting were fluid density (997 kg m^–3^), layer density (1100 kg m^–3^), and fluid viscosity (0.009 kg m^–1^ s^–1^). The following parameters were fitted while constrained to physically meaningful boundaries: layer viscosity (0.009–0.05 kg m^–1^ s^–1^), layer shear thickness (104–108 Pa), and layer thickness (10^–9^–2 × 10^–8^ m). The values of the hydrated protein layer (*T*_proteins_) were obtained by applying a similar fitting procedure.

#### Variable-Angle Spectroscopic Ellipsometry (VASE)

Au substrates (10 × 20 mm^2^) were immersed in a piranha solution (H_2_SO_4_:H_2_O_2_, 3:1) for 1 min, rinsed with ultrapure water and ethanol, and dried under a stream of N_2_. Then, they were incubated in an ethanolic solution of the different polymers (1 mg mL^–1^) for 1 h at ambient temperature. The samples were washed with ethanol to remove physisorbed polymer adsorbates and finally dried under a stream of N_2_.

The values of dry thickness (*T*_dry_) of the polymer adsorbates on Au substrates were measured with a variable-angle spectroscopic ellipsometer (M-2000F, Woollam Co., Inc., Lincoln, NE). The measurements were recorded within a range of wavelengths included between 400 and 850 nm using focusing lenses at 65° from the surface normal. Each data point resulted from an average of 20 measurements, and the obtained raw data were fitted with a bilayer model (Au and organic layer) using the analysis software WVASE32. The optical constants of the Au substrate (*n* and *k*) were experimentally obtained by measuring a freshly cleaned Au substrate with known thickness (200 nm). The organic adlayers were fitted using a Cauchy model approximated to the equation

$$n=A+\frac{B}{\lambda^{2}}$$

where *n* is the refractive index, λ is the wavelength, and *A* and *B* were assumed to be 1.45 and 0.01, respectively. The values of *T*_dry_ were used to calculate the grafting density (σ) by applying the equation

$$\sigma =\frac{\rho T_{dry}N_{a}}{M_{n}}$$

where ρ is the density of the dry polymer, *N*_A_ is the Avogadro number, and *M*_n_ is the number-average molecular weight of the adsorbate.

Polymer brush-functionalized substrates were then rehydrated with a PBS solution for 30 min. After that period, PBS was removed, and the substrates were incubated in 10% FHS for 1 h. The samples were washed with ultrapure water and dried under a stream of N_2_. The values of protein thickness (*T*_proteins_) were measured as described for *T*_dry_, using substrates with grafted polymer rehydrated for 1.5 h as reference. For all VASE experiments, mean values and standard deviations were calculated by using two replicates per sample and measuring four points on each of them.

#### UV–Visible Spectroscopy

UV–vis spectra were acquired on an Agilent Cary 60 spectrometer equipped with an 18-cell holder coupled to a Huber thermostat, using Suprasil quartz cuvettes (114-QS) from Hellma Analytics.

Turbidity measurements were acquired on an Agilent CARY 60 UV–vis spectrophotometer equipped with a thermoelectric cuvette holder with ethylene glycol as coolant, programmed to perform a temperature ramp of 1 °C/min. Quartz cuvettes with a 10 mm-optical path were used for the measurements, and transmittance was read at a fixed wavelength of 700 nm. All spectra were taken in ultrapure water at a polymer concentration of 5 mg mL^–1^.

The LCST values were determined from the inflection point of the sigmoidal fitting curve of the transmittance vs temperature plot using the following equation:

$$Trans=A-\frac{L}{1+e^{-k\left(T-LCST\right)}}$$

#### Contact Angle (CA)

Static water contact angles were determined using a manual goniometer (model 100, Ramé Hart Inc., USA) at room temperature and using ultrapure water (5 μL). Four measurements were conducted on each sample.

#### Synthesis of (monoethylene glycol)­vinyl ester (MEGVE)

MEGVE was synthesized following the procedure reported in the literature.^33^ A solution of 2-(2-methoxyethoxy)­acetic acid (10 mL, 88.0 mmol, 1 eq.), KOH (0.494 g, 8.8 mmol, 0.1 eq.), and Pd­(OAc)_2_ (0.988 g, 4.4 mmol, 0.05 eq.) in VAc (81 mL, 879.7 mmol, 10 eq.) were stirred at 60 °C overnight. The solution was filtered over Celite 545 and thoroughly washed with Et_2_O (30 mL). The excess of VAc and Et_2_O were removed under reduced pressure. The crude was purified through flash column chromatography using PE:EtOAC (7:3) as eluent mixture and then reduced to dryness using rotary evaporation to yield to a pale brown liquid. The monomer was further purified by vacuum distillation (∼1.1 mbar/90 °C) to yield MEGVE as a colorless oil (7.75 g, 48.4 mmol, 55% yield).

#### Synthesis of di­(ethylene glycol)­vinyl ester (DEGVE)

DEGVE was synthesized from [2-(2-methoxyethoxy)­ethoxy]­acetic acid (10 mL, 65.16 mmol, 1.0 eq.) following the procedure described for MEGVE. DEGVE was purified by flash column chromatography using PE:DCM (7:3) as eluent. The product was further purified by distillation (∼1.1 mbar/130 °C) (6.515 g, 31.90 mmol, 50% yield).

#### Synthesis of methoxyethoxy acrylate (MEGA)

K_2_CO_3_ (10.08 g, 72.9 mmol, 1.1 eq.) was dissolved in dry DMF (10 mL). Acrylic acid (5.00 mL, 72.9 mmol, 1.1 eq.) was slowly added to the solution at 0 °C under an Ar atmosphere, and the solution was stirred for 10 min. 2-Methoxyethoxymethyl chloride (7.57 mL, 66.3 mmol, 1 eq.) was added, and the solution was stirred under an Ar atmosphere, at 100 °C, for 3 h. The reaction mixture was extracted with LiCl_aq_ (5 w/v%)/EtOAc (3 × 200 mL), the organic fractions were collected and dried over Na_2_SO_4_, and the solvent was removed under vacuum. The crude was purified through flash column chromatography using DCM:EtOAc (95:5) as eluent to obtain the pure MEGA as a colorless oil (7.65 g, 47.7 mmol, 72% yield).

#### Synthesis of di­(ethylene glycol)­acrylate (DEGA)

Acrylic acid (25.0 mL, 364 mmol, 2 equiv), 2-(2-methoxyethoxy)­ethyl alcohol (21.4 mL, 182 mmol, 1 eq.), *p*-TsOH (3.462 g, 18.2 mmol, 0.1 eq.), and BHT (1.605 g, 7.3 mmol, 0.04 eq.) were dissolved in toluene (100 mL). The solution was stirred at 125 °C, using a Dean Stark apparatus overnight. The crude was purified by vacuum distillation (∼1.1 mbar/120 °C). The monomer was further purified through flash column chromatography using PE:EtOAc (7:3) as eluent to obtain the pure DEGA as a colorless oil (12.7 g, 72.8 mmol, 40% yield).

#### Synthesis of *O*-Ethyl-S-(1-methoxycarbony)­ethyldithiocarbonate (**1**)

Methyl 2-bromopropionate (5.001 g, 30 mmol, 1 eq.) was dissolved in dry MeOH (50 mL). Potassium *O*-ethyl dithiocarbonate (5.324 g, 33 mmol, 1.1 eq.) was added in 45 min to the solution at 0 °C. The solution was stirred 3 h at room temperature. The formed suspension was filtered under vacuum with a Büchner funnel and then concentrated under vacuum. The crude was extracted with DCM/H_2_O (5 × 50 mL), the organic fraction was dried over Na_2_SO_4_, and the solvent was removed under vacuum, to obtain the pure **1** (5.56 g, 26.7 mmol, 89% yield).

#### Synthesis of 2-(((Dodecylthio)­carbonothioyl)­thio)­propanoate (**2**)

**2** was synthesized following the procedure reported in the literature.^34^ Dodecanthiol (1.0 mL, 4.2 mmol, 1 eq.) was dissolved in chloroform (40 mL). TEA (0.870 g, 6.3 mmol, 1.5 eq.) was added, and the resulting solution was stirred for 1 h. CS_2_ (4.2 mL, 71 mmol, 17 eq.) was then added slowly. After 5 min, methyl 2-bromopropionate (0.699 g, 6.38 mmol, 1.5 eq.) was added, and the reaction mixture was stirred overnight. The mixture was then washed with HCl_aq_ (10%, 10 mL) and water (3 × 10 mL). The organic phase was collected and dried over Na_2_SO_4_, and all volatiles were removed under vacuum. The crude was purified through flash column chromatography using PE:EtOAc (7:3) as eluent to obtain the pure **2** as a yellow liquid (1.39 g, 3.8 mmol, 91% yield).

#### General Procedure for RAFT/RAFT-MADIX Polymerizations

Samples were taken during the reaction to monitor the kinetics of the polymerization. Each sample was diluted in CDCl_3_, and a ^1^H NMR spectrum was acquired. The signal of the polymerization solvent (DMF) or the signal of the added internal standard (DMF) was used to calibrate the integral of a selected peak. The conversion at time *t* (*C*_*t*_) was calculated using the following equation:

$$C_{t}\;\left(\%\right)=\frac{A_{0}-A_{t}}{A_{0}}\times 100$$

The theoretical molecular weights (*M*_n,th_) of the polymers were calculated based on the NMR conversion (*C*) using the following equation:

$$M_{n,th}=C\times DP_{T}\times MM_{mon}+MM_{CTA}$$

#### Synthesis of Poly­(monoethylene glycol)­vinyl ester-CTA (PMEGVE)-CTA)

MEGVE (1 g, 6.24 mmol, 100 eq.), **1** (12.1 mg, 0.0624 mmol, 1 eq.), AIBN (1.5 mg, 9.4 × 10^–3^ mmol, 0.15 eq.), and DMF (50 μL) were placed in a Schlenk tube. The solution was degassed for 30 min with Ar. The resulting solution was stirred and heated to 80 °C for 24 h. Samples were taken during the reaction for kinetic analysis, which was performed by ^1^H NMR spectroscopy. The polymer was dissolved in a small amount of DCM (1 mL) and precipitated Et_2_O (×5). The light-yellow oil was dried under vacuum at room temperature for 24 h. Conversion_MEGVE_ = 49%; *M*_n,th_ = 8.0 kDa.+

#### Synthesis of Poly­(methoxyethoxy acrylate)-CTA (PMEGA-CTA)

MEGA (1 g, 6.24 mmol, 60 equiv), **2** (37.9 mg, 0.104 mmol, 1 eq.), AIBN (1.7 mg, 10.4 × 10^–3^ mmol, 0.1 eq.), and DMF (898 μL, 50% v/v) were placed in a Schlenk tube. The solution was degassed 30 min with Ar. Later on, the polymerization mixture was stirred and heated to 70 °C for 3.5 h. Samples were taken during the reaction for kinetic analysis by ^1^H NMR spectroscopy. The obtained polymer was dissolved in a small amount of DCM (1 mL) and precipitated 5 times into Et_2_O. The product was finally dried under vacuum at room temperature for 24 h. Conversion_MEGA_ = 93%; *M*_n,th_ = 9.3 kDa.

#### Synthesis of Poly­(diethylene glycol)­acrylate-CTA (PDEGA-CTA)

PDEGA-CTA was prepared from DEGA (1 g, 5.74 mmol, 60 eq.) following the procedure described for PMEGA-CTA. Conversion_DEGA_ = 93%; *M*_n,th_ = 10.1 kDa.

#### Synthesis of poly­(diethylene glycol)­vinyl ester-CTA (PDEGVE-CTA)

PDEGVE-CTA was obtained by RAFT/MADIX polymerization of DEGVE (2 g, 9.79 mmol, 90 eq.) following the procedure described for PMEGA-CTA. The polymerization was stopped after 26 h. Conversion_DEGVE_ = 58%; *M*_n,th_ = 10.9 kDa.

#### General Procedure for CTA Removal

The xanthate and trithiocarbonate groups were removed to obtain thiol-terminated polymers. The same procedure was followed for POEGAs and POEGVEs. The procedure used for PMEGVE is reported as an example: PMEGVE-CTA (200 mg, 0.027 mmol, 1 eq.) was dissolved in THF (5 mL). BuNH_2_ (5.3 μL, 0.054 mmol, 2 eq.) was added to the solution under an Ar atmosphere. The reaction was stirred under an Ar atmosphere at room temperature overnight. Volatile compounds were removed under vacuum. The crude compound was dissolved in DCM, the insoluble component was removed by filtration, and the filtered solution was purified by dialysis (cutoff 1 kDa, gradient from DCM to EtOH); then the solvent was removed under vacuum to obtain the desired compound.

## Results and Discussion

We initially focused on the synthesis of POEGVEs and the corresponding POEGA counterparts, in both cases focusing on short side OEG segments with one and two ethylene glycol (EG) units.

Two different OEGVEs, (monoethylene glycol)­vinyl ester (MEGVE), which featured one EG unit, and (diethylene glycol)­vinyl ester (DEGVE), with two EG units, were synthesized by Pd-catalyzed transesterification between vinyl acetate and 2-(2-methoxyethoxy)­acetic acid or 2-[2-(2-methoxyethoxy)­ethoxy]­acetic acid, respectively (Figure S1 and Figures S3–S8).^33^

The corresponding acrylate monomers included methoxyethoxy acrylate (MEGA), which is isomeric to MEGVE, and di­(ethylene glycol)­acrylate (DEGA). The former was synthesized by coupling acrylic acid to 2-methoxyethoxymethyl chloride. Alternatively, DEGA was obtained by esterification of acrylic acid with 2-(2-methoxyethoxy)­ethyl alcohol catalyzed by *p*-toluenesulfonic acid (Figure S1 and Figures S3–S8).

Poly­(methoxyethoxy acrylate) (PMEGA) and poly­(diethylene glycol)­acrylate (PDEGA) were subsequently synthesized by reversible addition–fragmentation chain-transfer (RAFT) polymerization at 70 °C in DMF (50% v/v), using methyl 2-(((dodecylthio)­carbonothioyl)­thio)­propanoate as chain transfer agent (CTA) (Figures S2, S13, and S14) and 2,2′-azobis­(2-methylpropionitrile) (AIBN) as thermal radical initiator (Figure 1a, Table 1).

**Figure 1:**
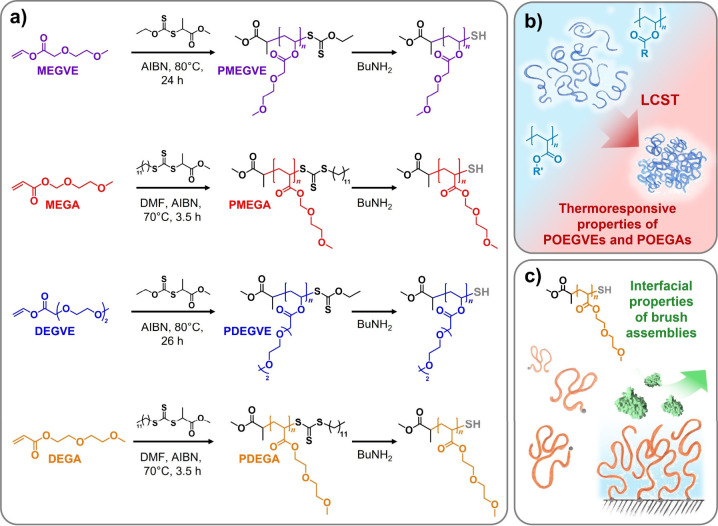
Synthesis of POEGAs and POEGVEs (a), analysis of their thermoresponsive properties (b), and evaluation of their interfacial physicochemical properties and biorepellent character when assembled on Au substrates as polymer brushes. (a) PMEGA and PDEGA were synthesized by RAFT polymerization, using methyl 2-(((dodecylthio)­carbonothioyl)­thio)­propanoate as CTA and AIBN as a source of radicals (70 °C in 50% v/v DMF solution). PMEGVE and PDEGVE were obtained by RAFT/MADIX polymerization using *O*-ethyl-*S*-(1-methoxycarbonyl)­ethyldithiocarbonate as CTA and AIBN as radical source (80 °C, bulk). Subsequent reaction with butylamine provided POEGA-SH and POEGVE-SH species, which were used for monitoring LCST and as adsorbates for Au-surface functionalization. (b) LCST monitoring was performed by UV–vis turbidity measurements of 5 mg mL^–1^ polymer solutions in water. (c) POEGA and POEGVE brushes were generated by grafting-to of SH-functional polymer adsorbates from methanolic solutions.

**Table 1:** Synthesis of POEGAs and POEGVEs by RAFT and RAFT/MADIX Polymerizations^a^

polymer	DP_target_	*t* (h)	*C* (%)^b^	*M*_n th_ ^c^ (kg mol^–1^)	*M*_n_ ^d^ (kg mol^–1^)	*M*_*w*_ ^d^ (kg mol^–1^)	*Đ* ^d^	DP_exp_ ^e^	*M*_n_ ^e^ (kg mol^–1^)	LCST (°C)
PMEGVE	100	24	49	7.8	9.1	13.6	1.5	55	8.9	64 ± 2
PDEGVE	90	3.5	58	10.9	11.3	17.0	1.5	60	12.2	86 ± 2
PMEGA	60	3.5	93	8.9	10.9	11.9	1.1	56	9.0	11 ± 2
PDEGA	60	26	93	9.7	10.5	12.5	1.2	51	8.9	45 ± 2

a Polymerization conditions: for POEGAs polymerizations were performed in DMF (50% v/v) at 70 °C with [M]:[CTA]:[AIBN] = DP_target_:1:0.1; for POEGVEs polymerizations were performed in bulk at 80 °C with [M]:[CTA]:[AIBN] = DP_target_:1:0.1.

b Conversion (mol %) estimated through ^1^H NMR.

c Theoretical *M*_n_ based on conversion calculated as *M*_n th_ = C × DP_target_ × MM_mon_ × MM_CTA_.

d Measured by SEC using DMF (with 1 g L^–1^ LiBr) as eluent (PMMA calibration).

e Estimated by ^1^H NMR of the purified compound as the ratio of the signals of the side chain and end group.

Simultaneously, poly­(monoethylene glycol)­vinyl ester (PMEGVE) and poly­(diethylene glycol)­vinyl ester (PDEGVE) were obtained by RAFT/macromolecular design by an interchange of xanthates (MADIX) process, in bulk at 80 °C, using *O*-ethyl-*S*-(1-methoxycarbony)­ethyldithiocarbonate as CTA and AIBN as initiator (Figures S2, S13, and S14).

In all cases, reaction parameters during RAFT and RAFT/MADIX processes were set to obtain POEGVEs and POEGAs all featuring a comparable degree of polymerization (DP_exp_, in Table 1) with *M*_n_ included between 9 and 11 kg mol^–1^, as measured by size exclusion chromatography (SEC). Dispersities (*Đ*) were centered around 1.1/1.2 for PMEGA and PDEGA, whereas (as expected) relatively higher values of 1.5 were obtained for PMEGVE and PDEGVE (Figures S15–S27).

The thermoresponsive properties of POEGVE and POEGA species were subsequently investigated using UV–vis turbidity measurements, monitoring the transmittance at 700 nm as a function of temperature (Figure 1b and Table 1). In order to exclude any contribution of chain-end functionalities—which presented markedly different polarity between POEGAs and POEGVEs (Figure 1a)—all polyvinyls were treated with butylamine, yielding thiol-terminating species in all cases (Figures S28–S37).

The temperature-induced transition for each polymer solution (5 mg mL^–1^) was recorded by using an automated system applying a temperature ramp of 1 °C min^–1^ during both heating and cooling cycles, yielding LCST values from the inflection point of the sigmoidal fitting of transmittance *vs* temperature profiles (Supporting Information).

Interestingly, both PMEGVE and PDEGVE exhibited significantly higher LCST values than their corresponding polyacrylate analogues, PMEGA and PDEGA (Figure 2). This difference was particularly pronounced for polymers bearing only a single EG unit in the side chain, namely, PMEGVE and PMEGA. Despite being isomeric polyvinyl species, these displayed markedly different thermoresponsive behavior, with a ΔLCST of approximately 53 °C. While PMEGVE remained fully soluble in water and showed no evidence of thermally induced aggregation up to 64 ± 2 °C, PMEGA was already insoluble in aqueous media at ambient temperature and exhibited an LCST of only 11 ± 2 °C.

**Figure 2:**
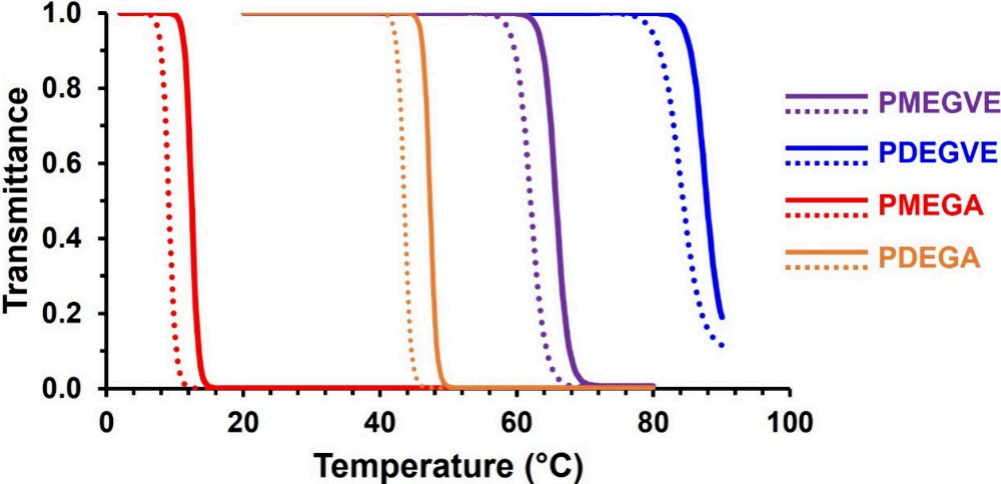
Normalized transmittance for POEGVE and POEGA solutions (5 mg mL^–1^) in water as a function of temperature. A temperature ramp of 1 °C min^–1^ was applied during heating (continuous lines) and cooling (dashed lines). LCST values were extracted from the inflection points of the heating and cooling profiles.

This trend was further supported by comparing the solution behavior of PDEGVE and PDEGA. Although these two polymers differ slightly in their chemical structure, both contain side chains comprising two EG units. In agreement with previous studies by O’Reilly and Dove,^33^ PDEGVE showed an LCST above 80 °C (86 ± 2 °C), whereas PDEGA underwent its phase transition at 45 ± 2 °C, corresponding to a ΔLCST of approximately 41 °C.

The significantly higher LCST observed for POEGVEs with respect to the POEGAs with comparable structures may be rationalized by the distinct connectivity of the ester functionality to the polymer backbone among the two different types of polyvinyls. In vinyl ester polymers, the backbone carbon is directly bonded to an oxygen atom of the ester group (−CH-O-C­(=O)-R), whereas in polyacrylates the backbone carbon is directly attached to the carbonyl carbon (−CH-C­(=O)-O-R). Consequently, POEGVEs feature an ether-type oxygen in immediate proximity to the carbon-carbon backbone, increasing the local polarity and hydrogen-bond-accepting capability near the main chain.^35^ This structural feature may enhance polymer-water interactions in addition to those provided by the OEG side chains, which are known to dominate the hydration of these polymers.^11,30,31,36^ Stronger overall hydration would stabilize the hydrated coil state and therefore require higher temperatures to trigger dehydration and phase transition,^37^ leading to the experimentally observed increase in LCST for POEGVEs relative to their POEGA counterparts.

The increased hydration by POEGVEs was further confirmed when these polyvinyls were applied to generate brush assemblies on model substrates. Brushes of POEGVEs and POEGAs were fabricated by assembling thiol-bearing polyvinyls on Au surfaces from ethanolic solutions (Supporting Information). For all the different polymer adsorbates the formation of brush assemblies showed a relatively fast kinetics, as evidenced by quartz crystal microbalance with dissipation (QCMD, Figure 3 and Figure S38). The dry thickness (*T*_dry_) of POEGVE and POEGA brushes in all cases was included between 1.7 and 2.0 nm, as measured by variable-angle spectroscopic ellipsometry (VASE), providing grafting density (σ) values that were comparable for all the different brush types—included between 0.10 and 0.12 chains cm^–2^ (Table 2). These values of σ agreed well with previous reports describing the assembly of brush layers through the grafting-to method in the case of polymer adsorbates presenting oligomeric side groups on each monomer unit.^25,38,39^ Comparison of VASE and QCMD data additionally enabled the estimation of the water content for each brush layer, which was expressed with their swelling ratio (SR), as (*T*_wet_ – *T*_dry_)/*T*_wet_ × 100 (Table 2). For all the brushes generated from polymer adsorbates presenting an LCST above ambient temperature (PMEGVE, PDEGVE, and PDEGA) SR values were >80%, confirming high hydration also when grafted on Au surfaces. In contrast, PMEGA brushes showed a significantly lower SR of 63 ± 9%, suggesting a lower tendency to associate water molecules within the polymer brush layer.

**Table 2:** Properties of POEGVE and POEGA Brush Assemblies Deposited on Au Substrates

	*T*_dry_ ^a^ (nm)	*T*_wet_ ^b^ (nm)	*T*_proteins_ ^b^ (nm)	*T*_proteins_ ^c^ (nm)	σ^d^ (chains nm^–2^)	SR^e^ (%)	CA (deg)
PMEGVE	1.9 ± 0.1	15.0 ± 0.1	0.1 ± 0.2	not meas.^f^	0.12 ± 0.01	87 ± 2	29 ± 2
PDEGVE	1.7 ± 0.1	11.0 ± 0.3	0.1 ± 0.1	not meas.^f^	0.10 ± 0.01	84 ± 4	29 ± 3
PMEGA	2.0 ± 0.1	5.6 ± 0.3	0.4 ± 0.2	2.4 ± 0.6	0.12 ± 0.01	63 ± 9	49 ± 1
PDEGA	1.8 ± 0.1	9.9 ± 0.1	0.3 ± 0.1	0.9 ± 0.5	0.11 ± 0.01	82 ± 2	37 ± 1

a *T*_dry_ was measured with VASE.

b *T*_wet_ and *T*_proteins_ were measured by the QCMD.

c Measured by VASE.

d Grafting density (σ), expressed as [chains nm^–2^], was calculated using the equation σ = *ρT*_dry_ *N*_A_ *M*_n_^–1^ where ρ is the density of the dry polymer layer (1.1 g cm^–3^), *T*_dry_ is the dry thickness measured by VASE, *N*_A_ is the Avogadro number, and *M*_n_ is the average molar mass of the adsorbate measured by SEC.

e Swelling ratio (SR) was estimated by applying SR = (*T*_wet_ – *T*_dry_)/ *T*_wet_ × 100.

f The amount of physisorbed protein was too low to be reliably determined.

**Figure 3:**
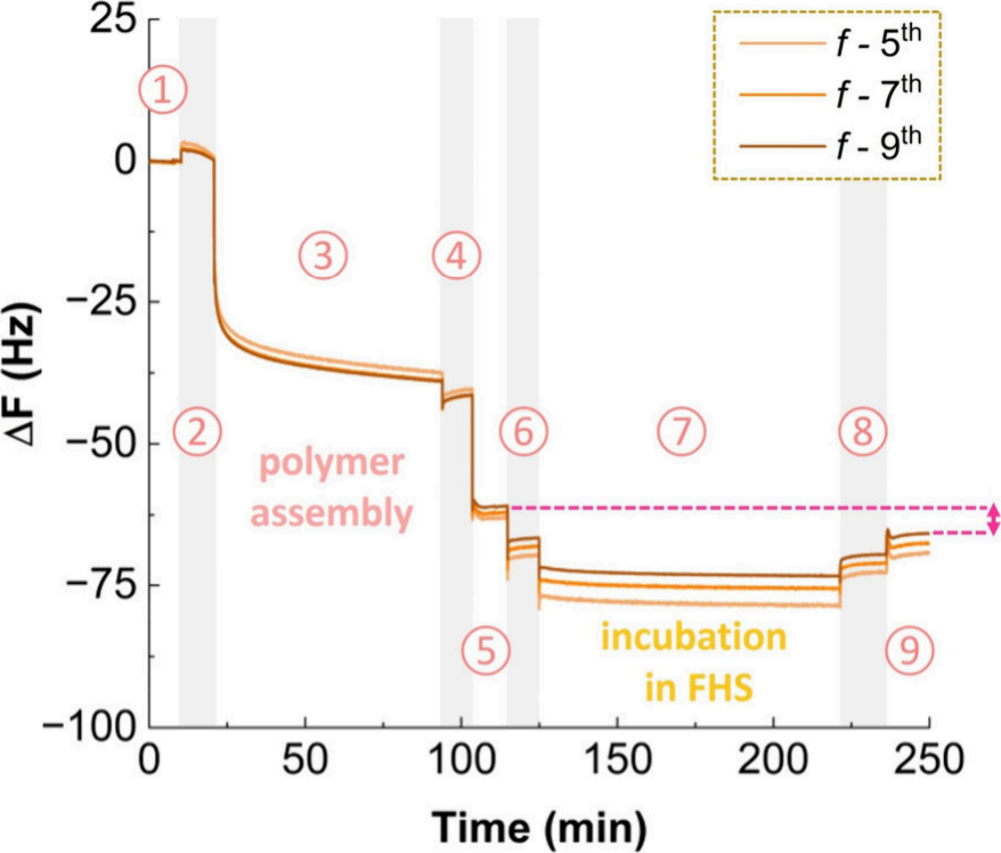
Representative QCMD sensograms reporting the variation of ΔF (overtones f-5^th^, f-7^th^, and f-9^th^) during the assembly of PDEGA adsorbates to form brushes on Au-coated sensors and the subsequent incubation in FHS. (1) Recording of baseline by incubation in ultrapure water, followed by flushing with methanol (2). (3) The sensor was subsequently subjected to 1 mg mL^–1^ PDEGA solution in ethanol, (4) followed by rinsing with pure ethanol and (5) ultrapure water. (6) Phosphate buffer saline (PBS) solution (pH = 7.4) was subsequently injected, (7) followed by 10% FHS solution. PDEGA brushes were finally rinsed (8) with PBS and (9) with ultrapure water.

These results were also confirmed while assessing the wettability of POEGVE and POEGA brushes by static water contact angle (CA). As highlighted in Table 2, PMEGVE and PDEGVE assemblies showed relatively low CA, which was ∼29° for both films. In contrast, significantly higher CAs of 49 ± 1° and 37 ± 1° were measured for PMEGA and PDEGA assemblies, respectively, highlighting how polyacrylate grafts showed a reduced tendency to hydrate when compared to their polyvinyl ester counterparts.

The enhanced hydration by PMEGVE and PDEGVE translated into an improved resistance toward contamination by serum proteins, as it was demonstrated by VASE and QCMD. After being incubated in 10% FHS solutions, ex situ VASE showed a nearly negligible amount of physisorbed proteins on both PMEGVE and PDEGVE brushes, with *T*_protein_ ∼ 0.1 nm. In contrast, protein contamination was more pronounced on PMEGA and PDEGA, with *T*_protein_ = 0.4 ± 0.2 nm and 0.3 ± 0.1 nm, respectively. The higher bioinertness toward FHS by PMEGVE and PDEGVE brushes was additionally confirmed by QCMD. As exemplarily reported in Figure 3 for PDEGA brushes, polyacrylate assemblies showed a non-negligible interaction with serum proteins, with especially PMEGA layers triggering the formation of 2.4 ± 0.6 nm-thick hydrated protein films (Table 2). In contrast, both PMEGVE and PDEGVE grafts efficiently prevented contamination by serum proteins (Supporting Information and Table 2), suggesting how their enhanced tendency to associate water molecules was also mirrored by an improved antibiofouling character.

## Conclusions

POEGVEs emerge as a promising platform for the generation of polymer brush biointerfaces displaying enhanced hydration and excellent bioinertness compared with their polyacrylate analogues. Although OEG-functionalized polymers have traditionally relied on poly­(meth)­acrylate backbones, the present study demonstrates that the corresponding poly­(vinyl ester) architectures provide distinct advantages arising from their backbone connectivity. The RAFT/MADIX process applied for the synthesis of POEGVEs provides polymers with larger dispersities compared to those of the corresponding POEGMAs obtained through RAFT polymerization and implies longer reaction times. However, the direct comparison of POEGVEs bearing short OEG side chains with polyacrylate counterparts demonstrates that vinyl ester polymers exhibit markedly higher LCST values in aqueous solution, which are consistent with stronger polymer-water interactions. When assembled as polymer brush layers on model Au substrates, this enhanced hydration translated into higher swelling, increased wettability, and significantly improved resistance toward nonspecific protein adsorption from FHS.

These findings highlight how modulation of polymer backbone chemistry can profoundly influence hydration, thermoresponsive behavior, and interfacial performance. More generally, POEGVEs emerge as a previously overlooked yet highly promising polymer platform for the design of thermoresponsive and antibiofouling biointerfaces, opening new opportunities in materials science and biomaterials’ development.

## Supplementary Material



## Notes

SEC data, NMR spectra, QCMD sensograms (PDF)
