# Supplementary material for: Molecular Design of Thermoresponsive Poly(oligoethylene glycol)vinyl Esters for the Generation of Polymer Brush-Based Biointerfaces
Source: ACS Polym Au. 2026 May 8;6(4):1116–23. doi: 10.1021/acspolymersau.6c00053 (PMC13474339; doi:10.1021/acspolymersau.6c00053)
Supplement: Supplementary file 1 [file lg6c00053_si_001.pdf]

# Supporting Information

## Molecular Design of Thermoresponsive Poly(Oligoethylene Glycol)Vinyl Esters for the Generation of Polymer Brush-Based Biointerfaces

*Francesco M. Ara,[1] Rachele Foladore,[1] Alessandro Magro,[1] Nicola Scapin,[1] Daniele Rizzi,[1] Alessandro Bertolo,[1] Francesca Lorandi,[1] Edmondo M. Benetti[1]*

[1]Laboratory for Macromolecular and Organic Chemistry, Department of Chemical Sciences, University of Padova, via Marzolo 1, 35131 Padova, Italy.

1 Abbreviations .......... 2
2 Synthesis .......... 3
2.1 MEGVE .......... 3
2.2 DEGVE .......... 4
2.3 MEGA .......... 5
2.4 DEGA .......... 6
2.5 O-ethyl-S-(1-methoxycarbony)ethyldithiocarbonate .......... **Error! Bookmark not defined.**
2.6 2-(((dodecylthio)carbonothioyl)-thio)propanoate .......... **Error! Bookmark not defined.**
3 Polymerizations .......... 9
3.1 Thermal RAFT polymerization .......... 9
3.1.1 PMEGVE-CTA .......... 10
3.1.2 PMEGA-CTA .......... 11
3.1.3 PDEGA-CTA .......... 13
3.1.4 PDEGVE-CTA .......... 14
3.2 Post-polymerization modification .......... 16
3.2.1 PMEGVE-SH .......... 17
3.2.2 PMEGA-SH .......... 18
3.2.3 PDEGA-SH .......... 19
3.2.4 PDEGVE-SH .......... 20
3.3 QCMD Sensograms .......... 21

# 1 Abbreviations

| | |
|---|---|
| AIBN: | 2,2′-azobis(2-methylpropionitrile) |
| Aq: | Aqueous solution |
| BHT: | Butylated hydroxytoluene |
| Bu: | Buthyl |
| CA: | Contac angle |
| CTA: | Chain Transfer Agent |
| DCM: | Dichloromethane |
| DEGA: | Diethyleneglycol acrylate |
| DEGVE: | Diethyleneglycol vinyl ester |
| DMF: | N,N-dimethylformamide |
| $DP_t$: | Target Degree of Polymerization |
| Et: | Ethyl |
| $Et_2O$: | Diethyl ether |
| EtOAc: | Ethyl acetate |
| EtOH: | Ethanol |
| Hex: | Hexane |
| LCST: | Lower Critical Solution Temperature |
| Me: | Methyl |
| MEGA: | Methoxyethoxy acrylate |
| MEGVE: | Monoethyleneglycol vinyl ester |
| MEHQ: | Methylhydroquinone |
| MeOH: | Methanol |
| MM: | Molecular Mass |
| NMR: | Nuclear Magnetic Resonance |
| OAc: | Acetate |
| o.n.: | over night |
| PBS: | phosphate buffered saline |
| PE: | Petroleum ether |
| PI-RAFT: | Photo Iniferter RAFT |
| PMMA: | Poly(methyl methacrylate) |
| p-TsOH: | p-Toluenesulfonic acid monohydrate |
| QCMD: | Quartz Crystal Microbalance with Dissipation |
| RAFT: | Reversible Addition-Fragmentation chain Transfer |
| r.t.: | Room temperature |
| SEC: | Size Exclusion Chromatography |
| TLC: | Thin-layer chromatography |
| TEA: | Triethylamine |
| THF: | Tetrahydrofuran |
| Tol: | Toluene |
| VAc: | Vinyl acetate |
| VASE: | Variable Angle Spectroscopic Ellipsometry |

# 2 Synthesis

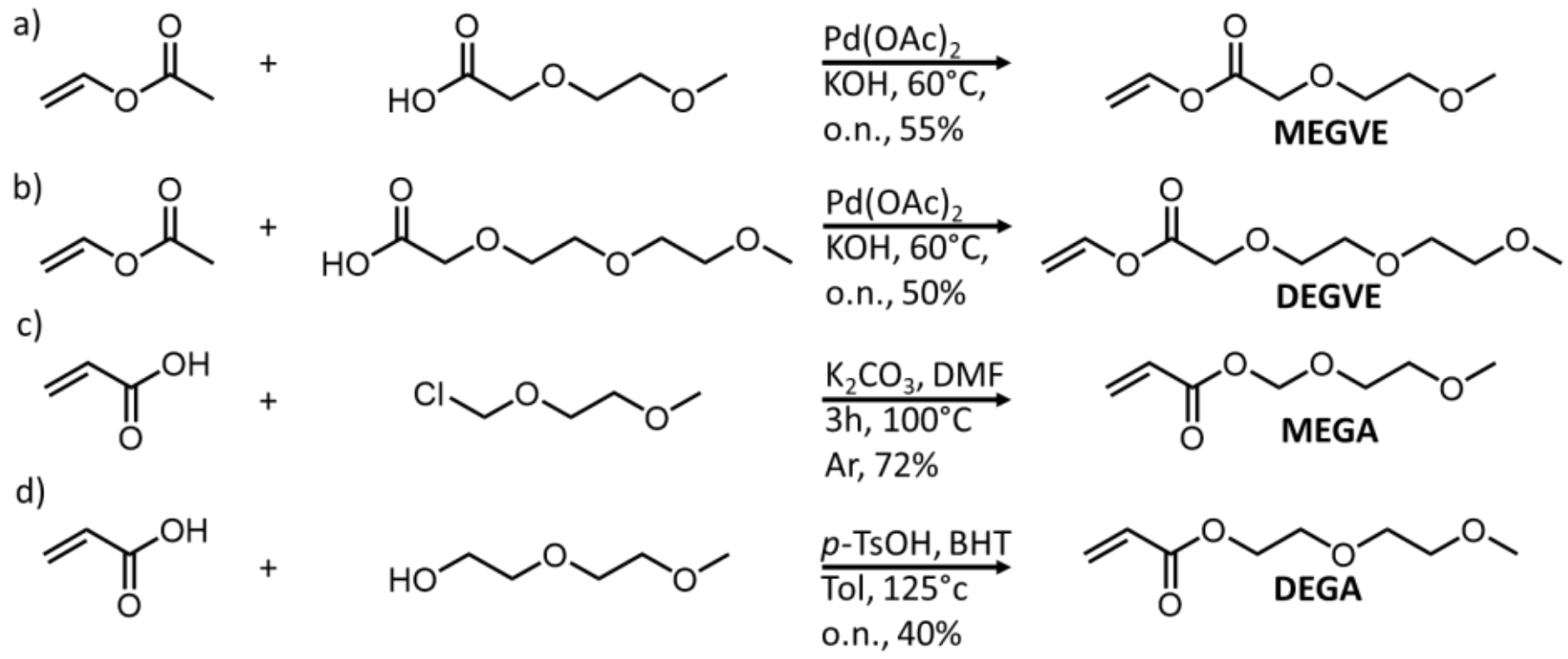


**Figure S1:** Synthetic scheme for the synthesis of monomers. **a)** MEGVE, **b)** DEGVE, **c)** MEGA and **d)** DEGA.

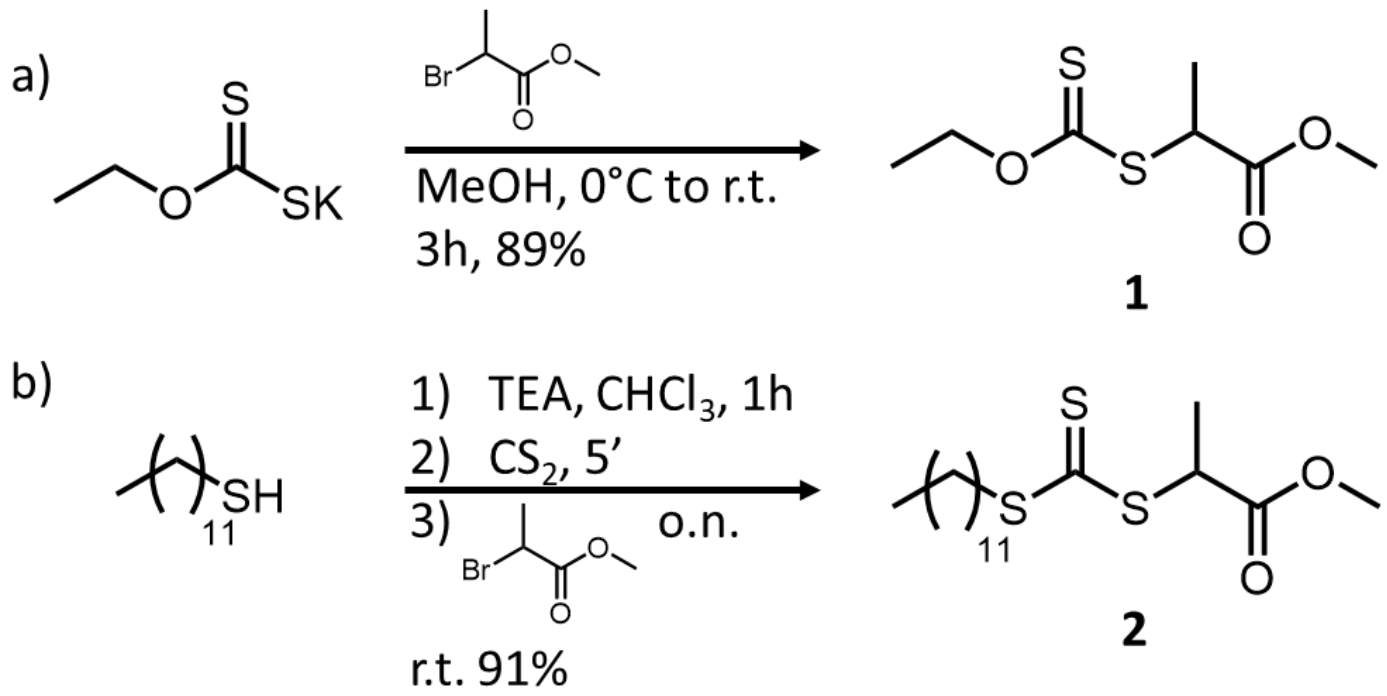


**Figure S2:** Synthetic scheme for the synthesis of CTAs. **a)** O-ethyl-S-(1-methoxycarbony)ethyldithiocarbonate (**1**) and **b)** 2-(((dodecylthio)carbonothioyl)-thio)propanoate (**2**).

## 2.1 MEGVE

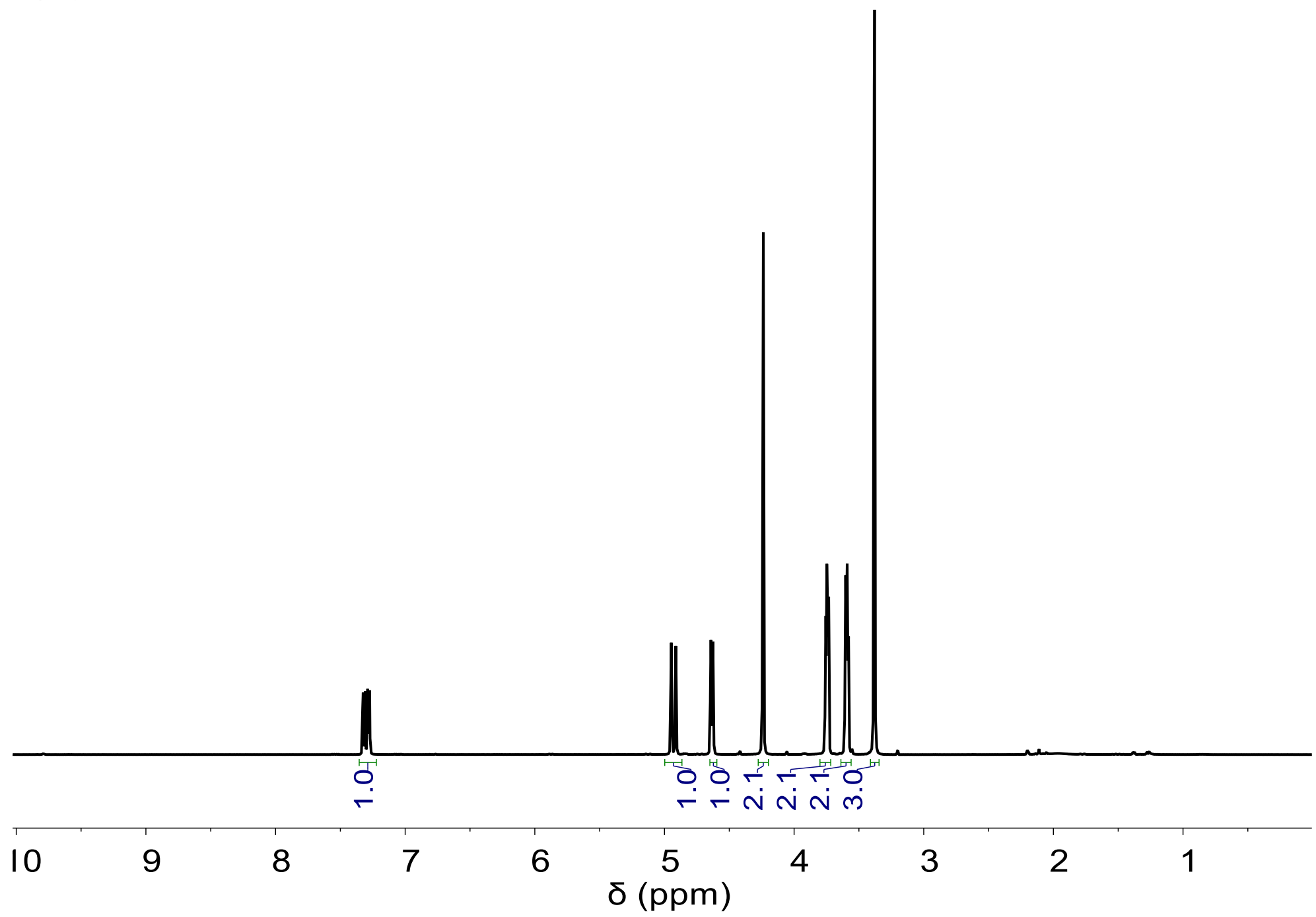


**Figure S3:** $^{1}$H-NMR spectrum (400MHz, $CDCl_3$) of MEGVE. δ 7.30 (dd, *J* = 13.9, 6.2 Hz, 1H, C=C*H*-), 4.93 (d, *J* = 13.9 Hz, 1H, C=C*H₂*), 4.63 (d, *J* = 6.2 Hz, 1H, C=C*H₂*), 4.24 (s, 2H, COO-C*H₂*-O), 3.74 (t, *J* = 4.2 Hz, 2H, O-C*H₂*-), 3.59 (t, *J* = 4.2 Hz, 2H, O-C*H₂*-), 3.38 (s, 3H, -C*H₃*).

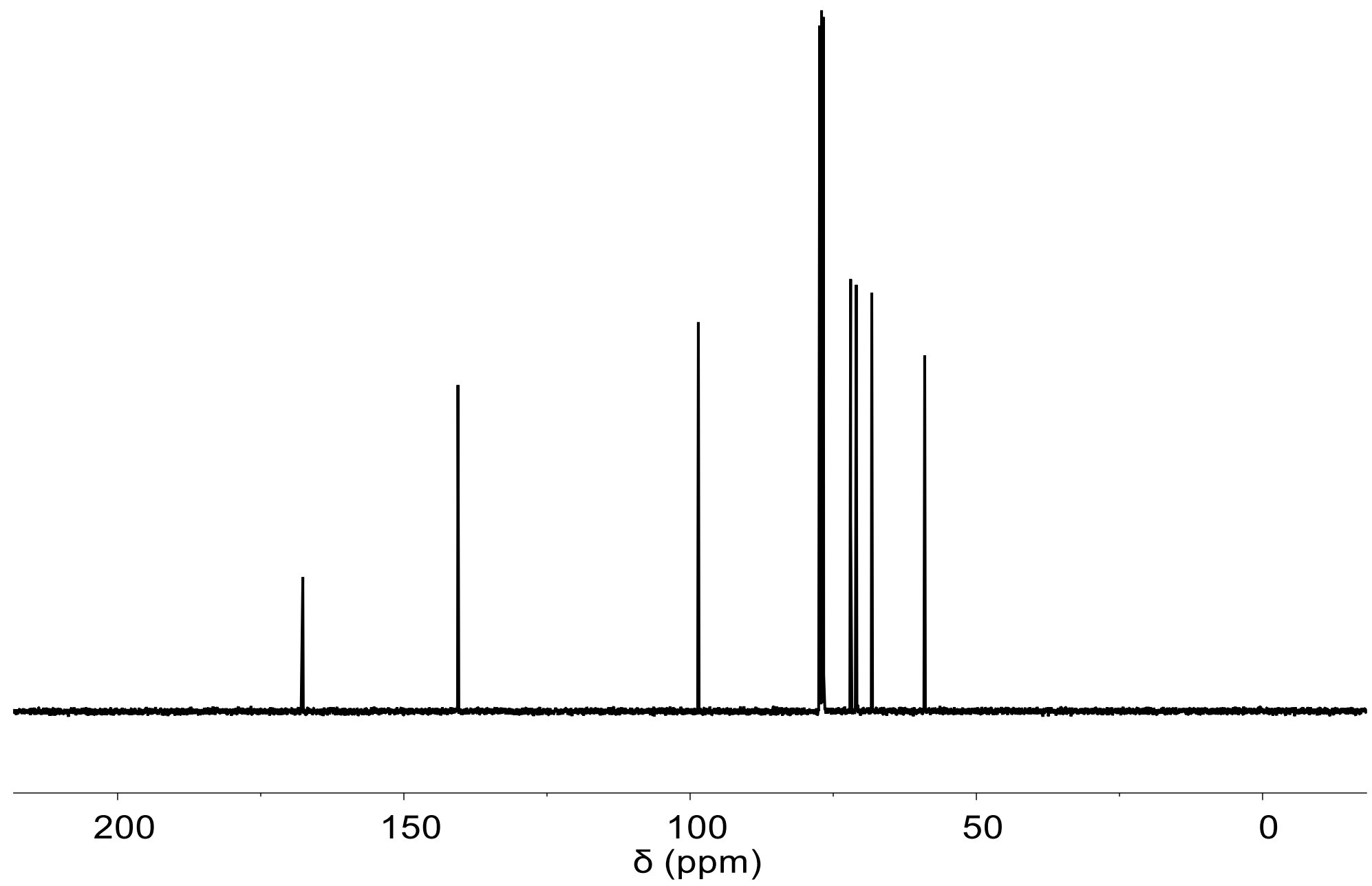


**Figure S4:** $^{13}$C-NMR spectrum (101MHz, $CDCl_3$) of MEGVE. δ 167.65 (*C*=O), 140.55 (O-*C*H=$CH_2$), 98.56 (CH=*C*$H_2$), 71.96 (-*C*$H_2$-O), 70.98 (O-*C*$H_2$-), 68.27 ($CO_2$-*C*$H_2$-O), 59.02 (-*C*$H_3$).

## 2.2 DEGVE

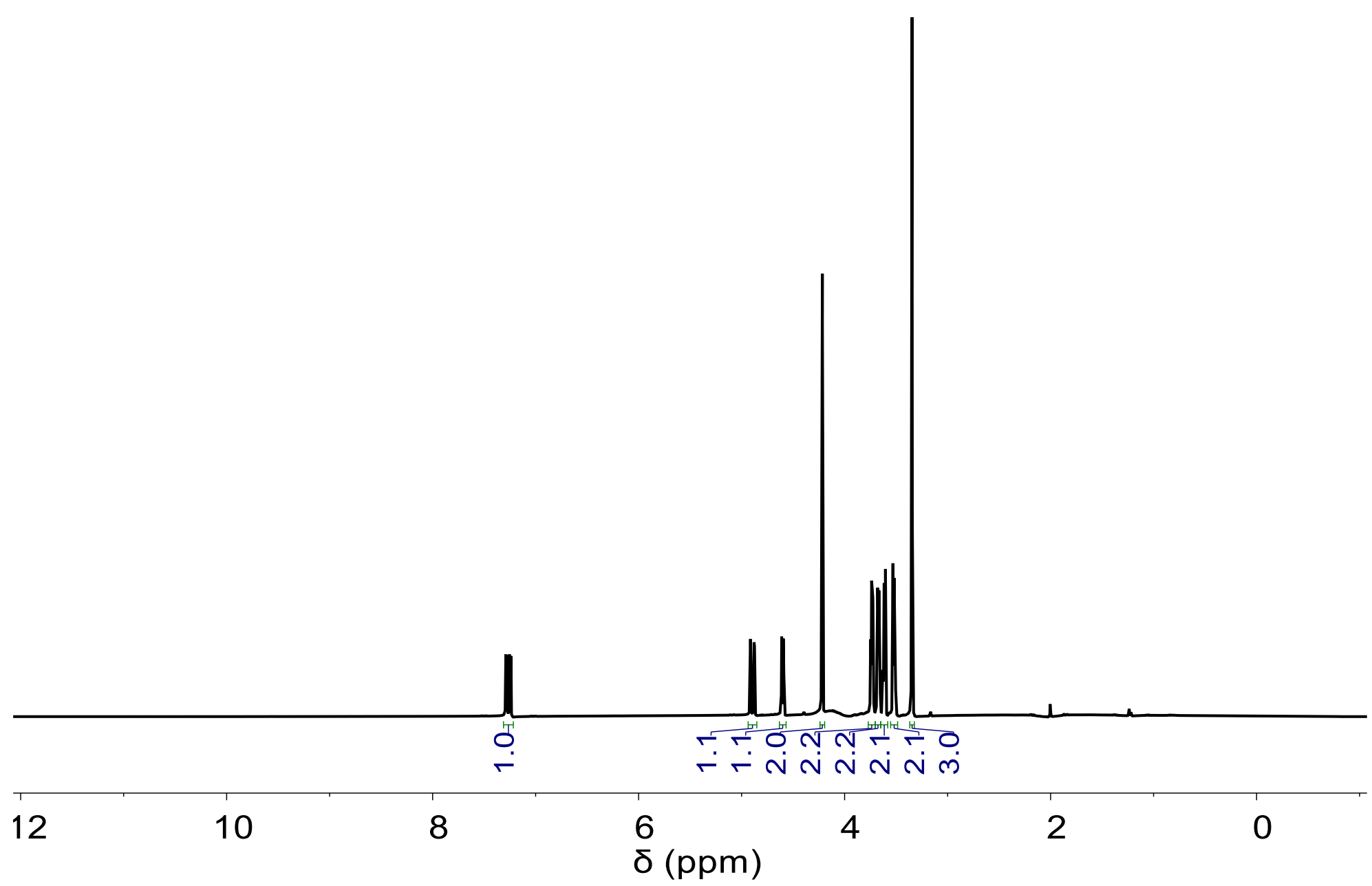


**Figure S5:** $^{1}$H-NMR spectrum (400MHz, $CDCl_3$) of DEGVE. δ 7.26 (dd, *J* = 13.9, 6.2 Hz, 1H, C=C*H*-), 4.89 (dd, *J* = 13.9, 1.8 Hz, 1H, C=C*$H_2$*), 4.60 (dd, *J* = 6.3, 1.8 Hz, 1H, C=C*$H_2$*), 4.22 (s, 2H, COO-C*$H_2$*-O), 3.76 – 3.71 (m, 2H, O-C*$H_2$*-), 3.69 – 3.65 (m, 2H, O-C*$H_2$*-), 3.63 – 3.59 (m, 2H, O-C*$H_2$*-), 3.55 – 3.49 (m, 2H, O-C*$H_2$*-), 3.34 (s, 3H, -C*$H_3$*).

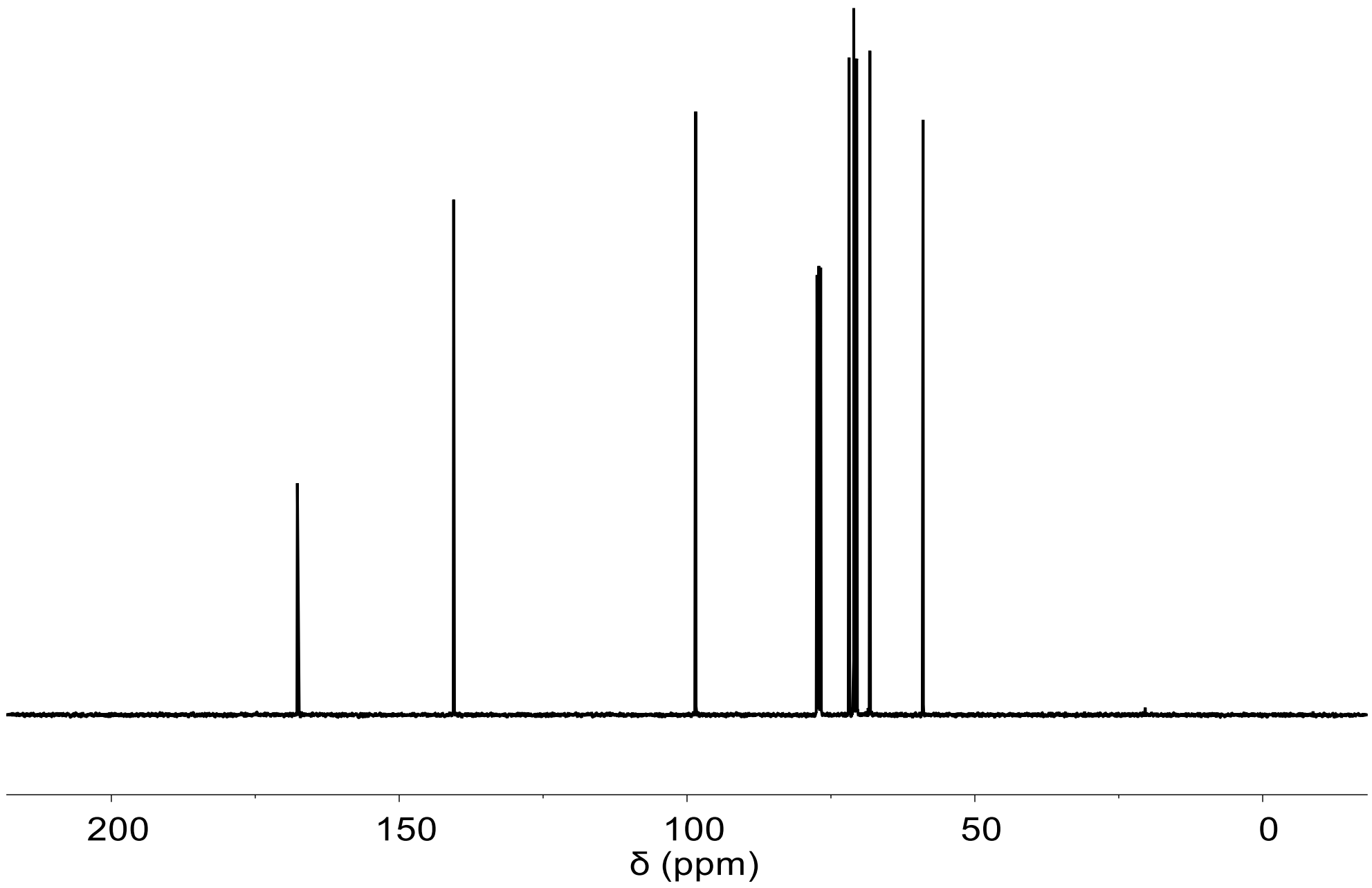


**Figure S6:** $^{13}$C-NMR spectrum (101MHz, $CDCl_3$) of DEGVE. δ 167.69 (*C*=O), 140.53 (O-*C*H=$CH_2$), 98.49 (CH=*C*$H_2$), 71.85 (-*C*$H_2$-O), 71.03 (-*C*$H_2$-O), 70.70 (-*C*$H_2$-O), 70.51 (-*C*$H_2$-O), 68.24 ($CO_2$-*C*$H_2$-O), 58.99 (-*C*$H_3$).

## 2.3 MEGA

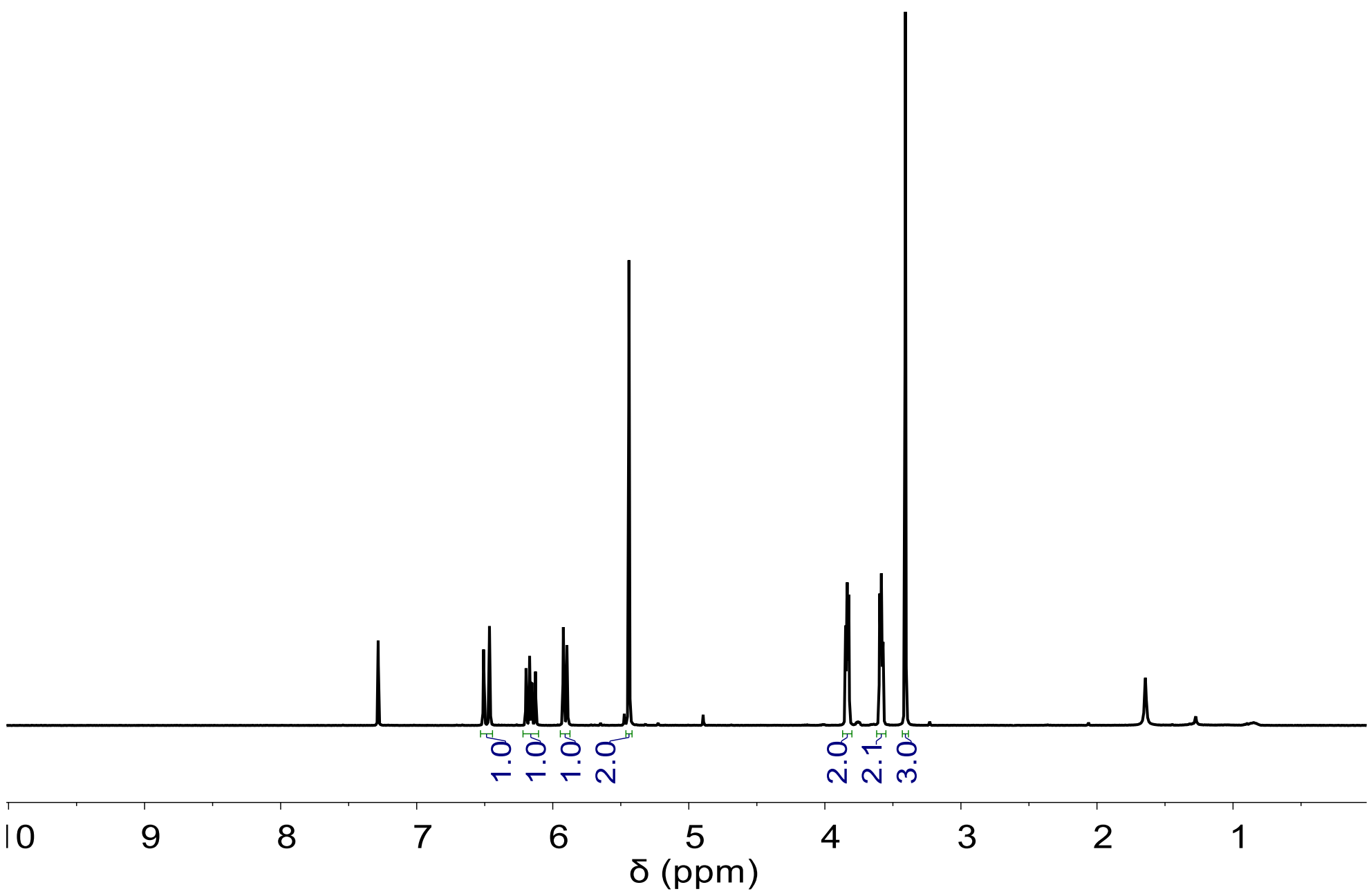


**Figure S7:** $^{1}$H-NMR spectrum (400MHz, $CDCl_3$) of MEGA. δ 6.49 (d, *J* = 17.3 Hz, 1H, C=C*$H_2$*), 6.16 (dd, *J* = 17.3, 10.5 Hz, 1H, C=C*H*-), 5.91 (d, *J* = 10.4 Hz, 1H, C=C*$H_2$*), 5.44 (s, 2H, O-C*$H_2$*-O), 3.84 (t, *J* = 4.4 Hz, 2H, O-C*$H_2$*-), 3.59 (t, *J* = 4.4 Hz, 2H, O-C*$H_2$*-), 3.41 (s, 3H, -$CH_3$).

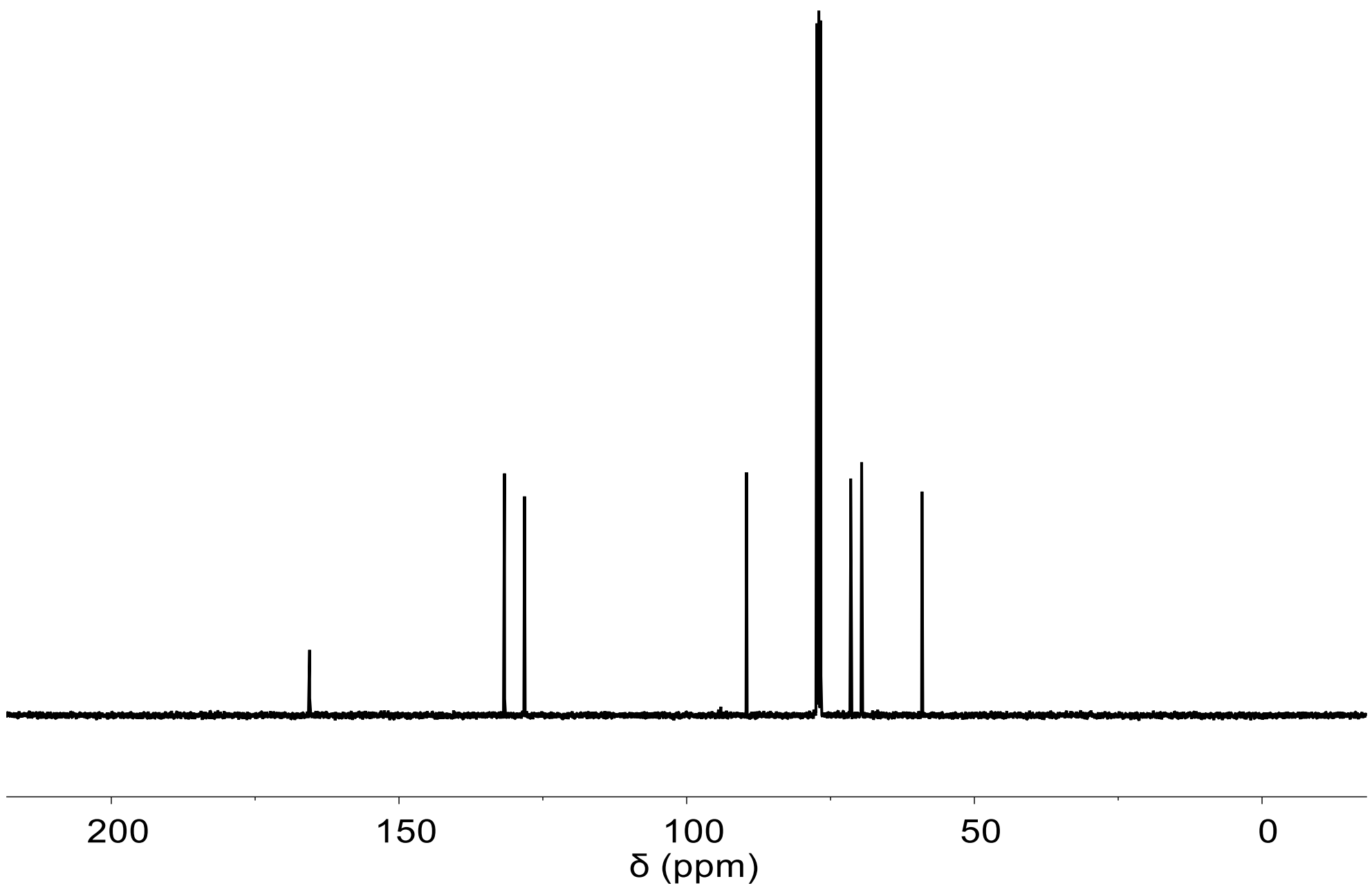


**Figure S8:** $^{13}$C-NMR spectrum (101 MHz, $CDCl_3$) of MEGA. δ 165.53 (*C*=O), 131.66 (CH=*C*$H_2$), 128.18 (O-*C*H=$CH_2$), 89.60 (O-*C*$H_2$-O), 71.50 (-*C*$H_2$-O), 69.59 (O-*C*$H_2$-), 59.09 (-*C*$H_3$).

## 2.4 DEGA

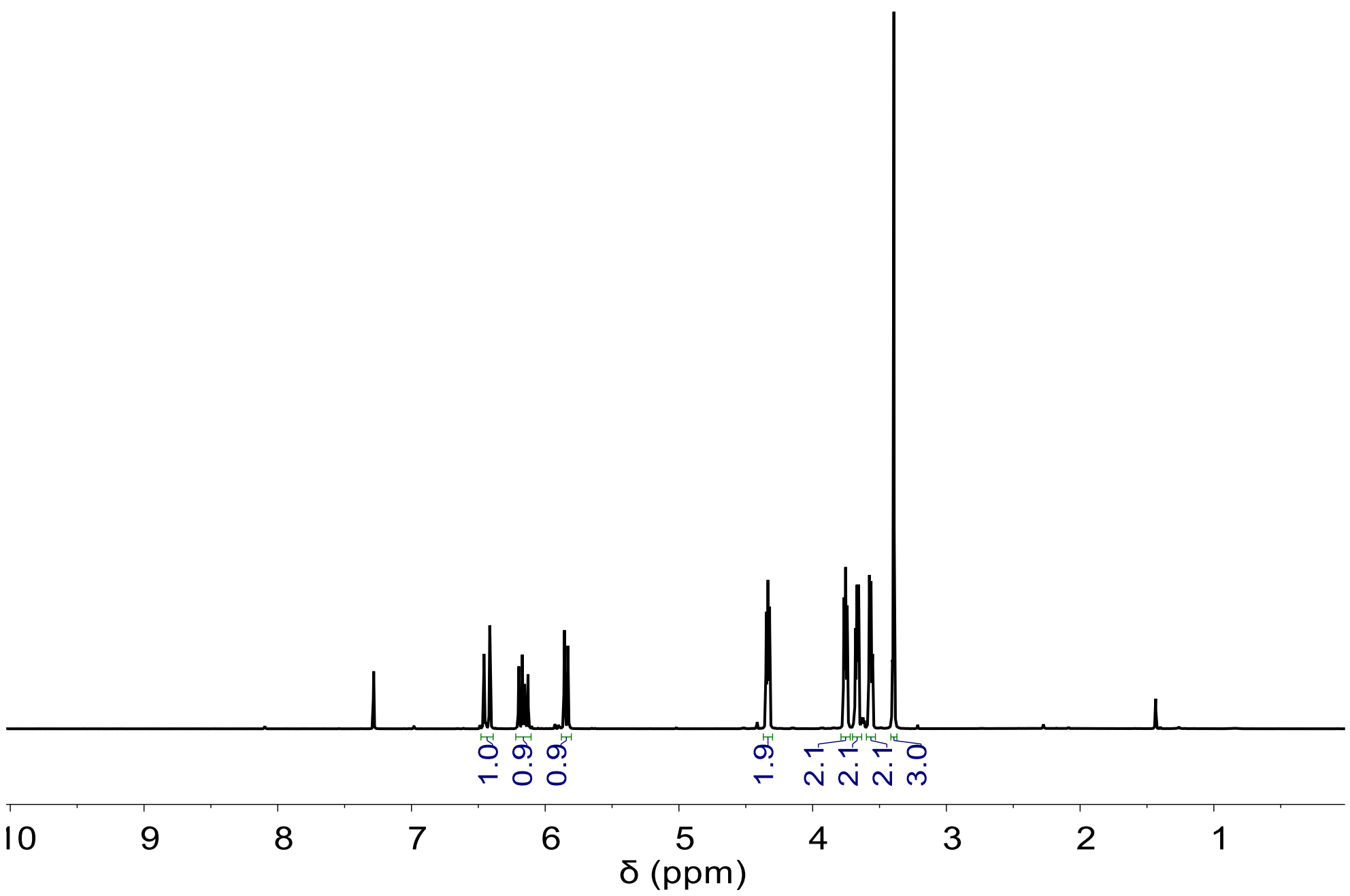


**Figure S9:** $^{1}$H-NMR spectrum (400MHz, $CDCl_3$) of DEGA. δ 6.43 (dd, *J* = 17.3, 1.3 Hz, 1H, C=$C_{H2}$), 6.16 (dd, *J* = 17.3, 10.4 Hz, 1H, C=C*H*-), 5.84 (dd, J = 10.4, 1.3 Hz, 1H, C=C*$H_2$*), 4.33 (t, *J* = 4.9 Hz, 2H, O-C*$H_2$*-), 3.75 (t, *J* = 4.8 Hz, 2H, O-C*$H_2$*-), 3.67 (t, *J* = 4.9 Hz, 2H, O-C*$H_2$*-), 3.56 (t, *J* = 4.8 Hz, 2H, O-C*$H_2$*-), 3.39 (s, 3H, -C*$H_3$*).

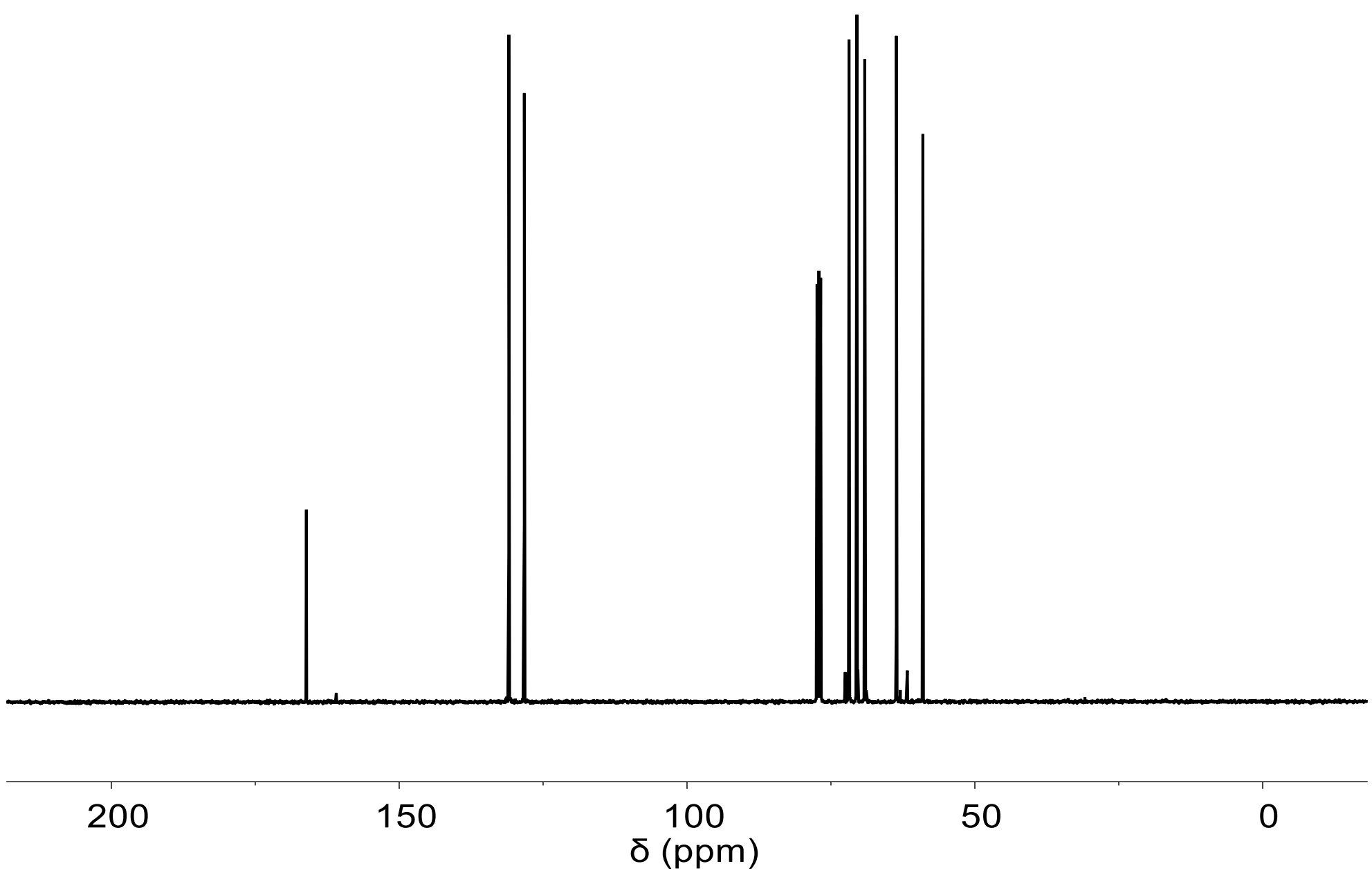


**Figure S10:** $^{13}$C-NMR spectrum (101 MHz, $CDCl_3$) of DEGA. δ 166.13 (*C*=O), 130.95 (CH=*C*$H_2$), 128.26 (O-*C*H=$CH_2$), 71.86 (-*C*$H_2$-O), 70.50 (-*C*$H_2$-O), 69.12 (-*C*$H_2$-O), 63.63 (-*C*$H_2$-O), 59.03 (-*C*$H_3$).

## 2.5 2-(((dodecylthio)carbonothioyl)-thio)propanoate

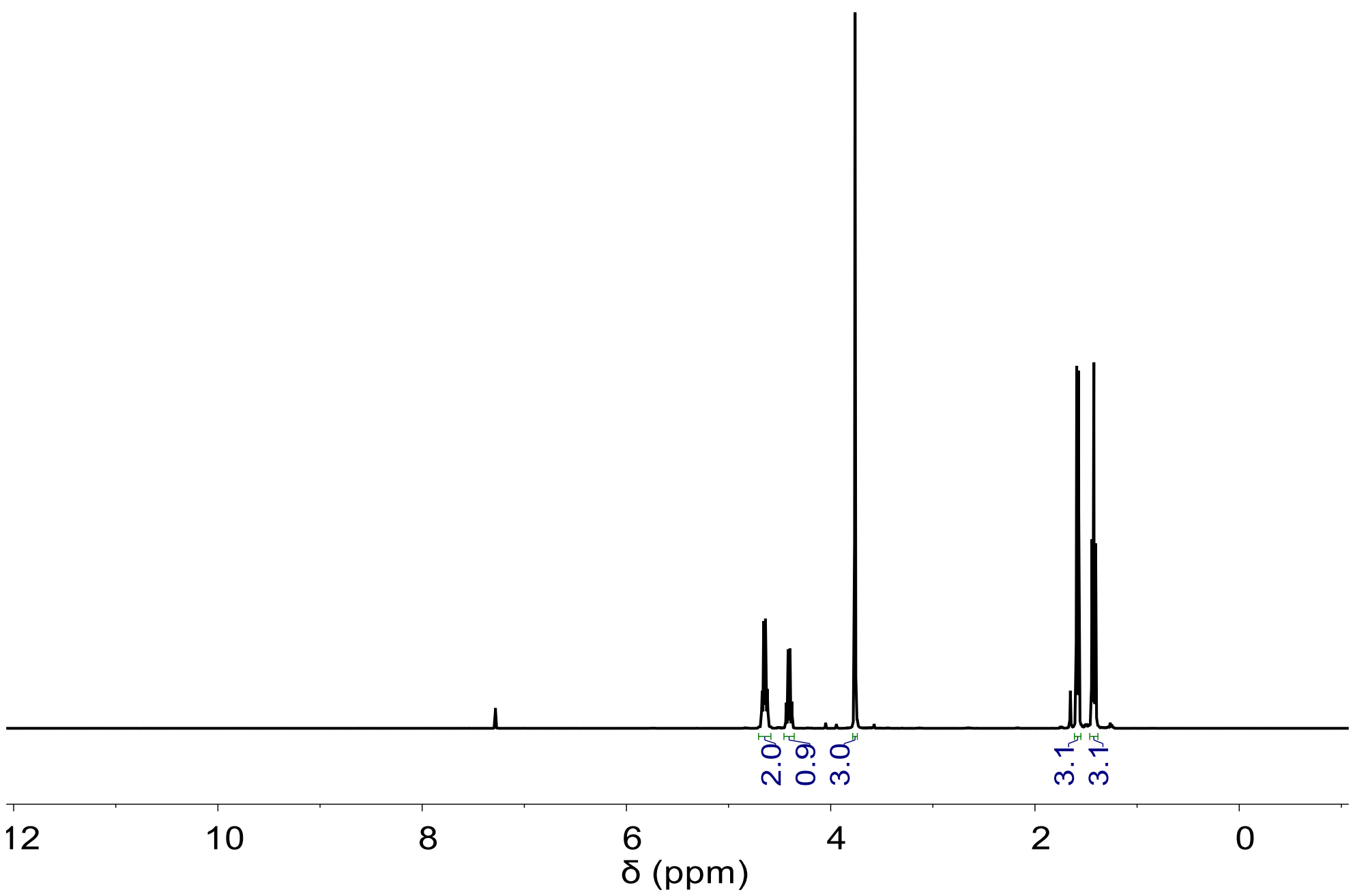


**Figure S11:** $^{1}$H-NMR spectrum (400MHz, $CDCl_3$) of **1**. δ 4.65 (qd, *J* = 7.1, 1.6 Hz, 2H, -C*$H_2$*-), 4.41 (q, *J* = 7.4 Hz, 1H, -C*H*-), 3.76 (s, 3H, O-C*$H_3$*), 1.58 (d, *J* = 7.4 Hz, 3H, CH-C*$H_3$*), 1.42 (t, *J* = 7.1 Hz, 3H, $CH_2$-C*$H_3$*).

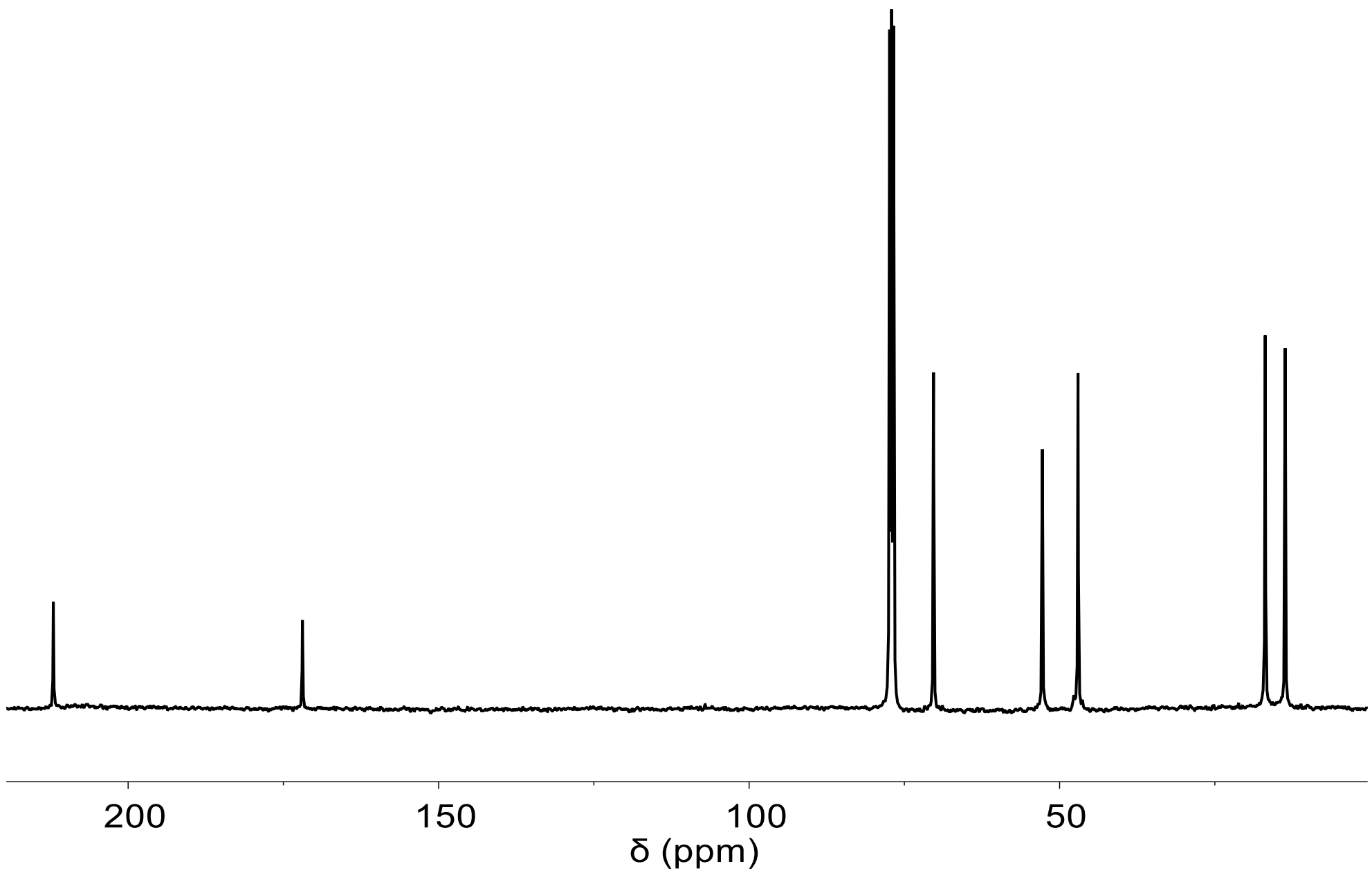


**Figure S12:** $^{13}$C-NMR spectrum (101 MHz, $CDCl_3$) of **1**. δ 212.04 (*C*=S), 171.93 (*C*=O), 70.30 (-*C*$H_2$-), 52.78 (O-*C*$H_3$), 47.03 (-*C*H-), 16.90 (-CH-*C*$H_3$), 13.68 (-$CH_2$-*C*$H_3$).

## 2.6 2-(((dodecylthio)carbonothioyl)-thio)propanoate

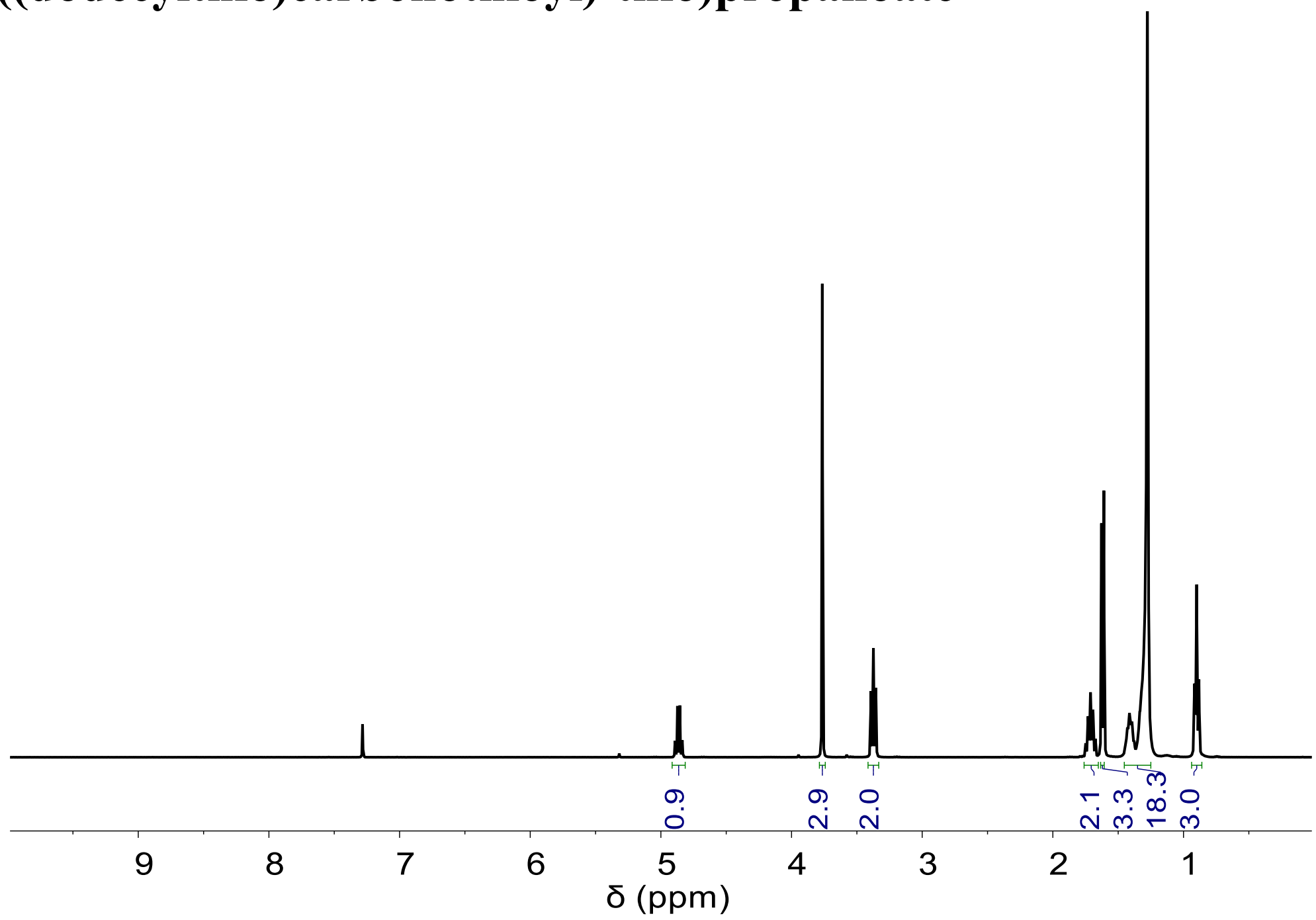


**Figure S13:** $^{1}$H-NMR spectrum (400MHz, $CDCl_3$) of **2**. δ 4.86 (q, $J$ = 7.4 Hz, 1H, -C*H*-), 3.76 (s, 3H, O-C*$H_3$*), 3.37 (t, $J$ = 7.4 Hz, 2H, S-C*$H_2$*-), 1.71 (p, $J$ = 7.4 Hz, 2H, -C*$H_2$*-$CH_2$-S), 1.62 (d, $J$ = 7.4 Hz, 3H, CH-C*$H_3$*), 1.47 – 1.25 (m, 18H, -$CH_2$-), 0.90 (t, $J$ = 6.7 Hz, 3H, $CH_2$-C*$H_3$*).

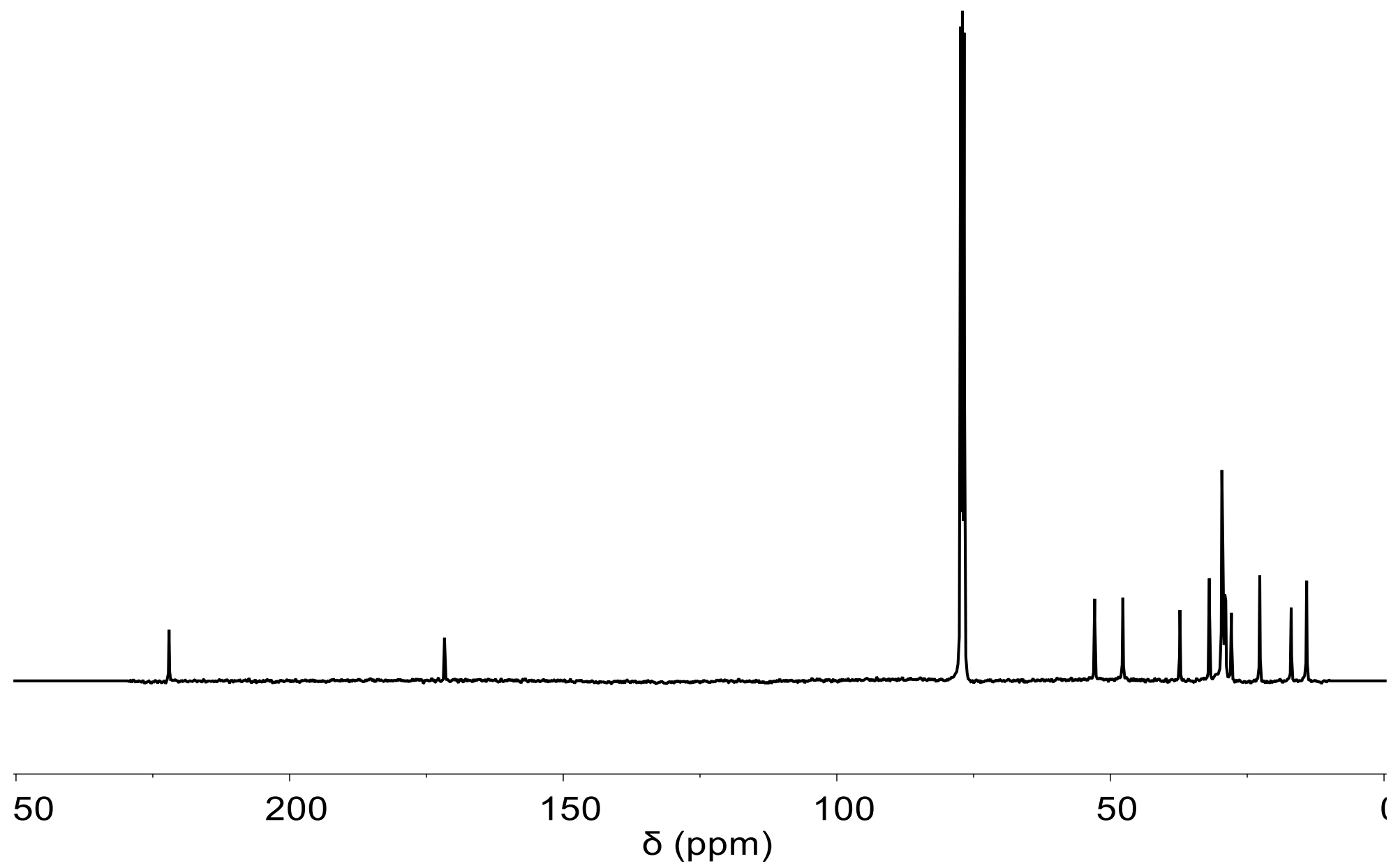


**Figure S14:** $^{13}$C-NMR spectrum (101 MHz, $CDCl_3$) of **2**. δ 222.03 (*C*=S), 171.69 (*C*=O), 52.88 (O-$CH_3$), 47.73 (-*C*H-), 37.30 (S-$CH_2$-), 31.93 (-$CH_2$-), 29.64 (-$CH_2$-), 29.63 (-$CH_2$-), 29.55 (-$CH_2$-), 29.45 (-$CH_2$-), 29.35 (-$CH_2$-), 29.10 (-$CH_2$-), 28.92 (-$CH_2$-), 27.89 (-$CH_2$-), 22.71 (-$CH_2$-), 16.96 (-CH-$CH_3$), 14.14 (-$CH_2$-$CH_3$).

# 3 Polymerizations

## 3.1 Thermal RAFT polymerization

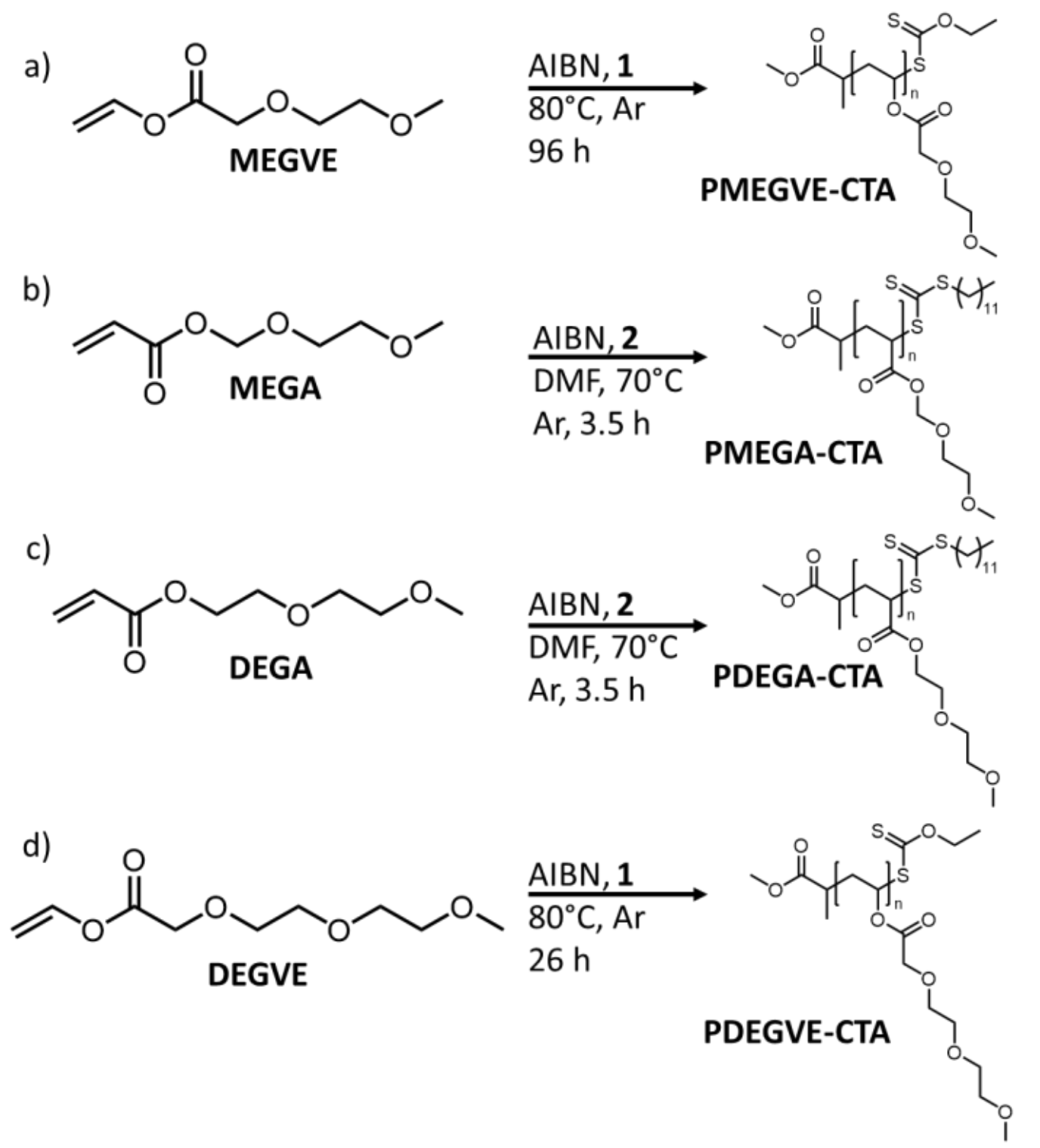


**Figure S15:** Polymerizations of OEGVEs. and OEGAs. **a)** PMEGVE-CTA, **b)** PDEGVE-CTA, **c)** PMEGA-CTA and **d)** PDEGA-CTA.

## 3.1.1 PMEGVE-CTA

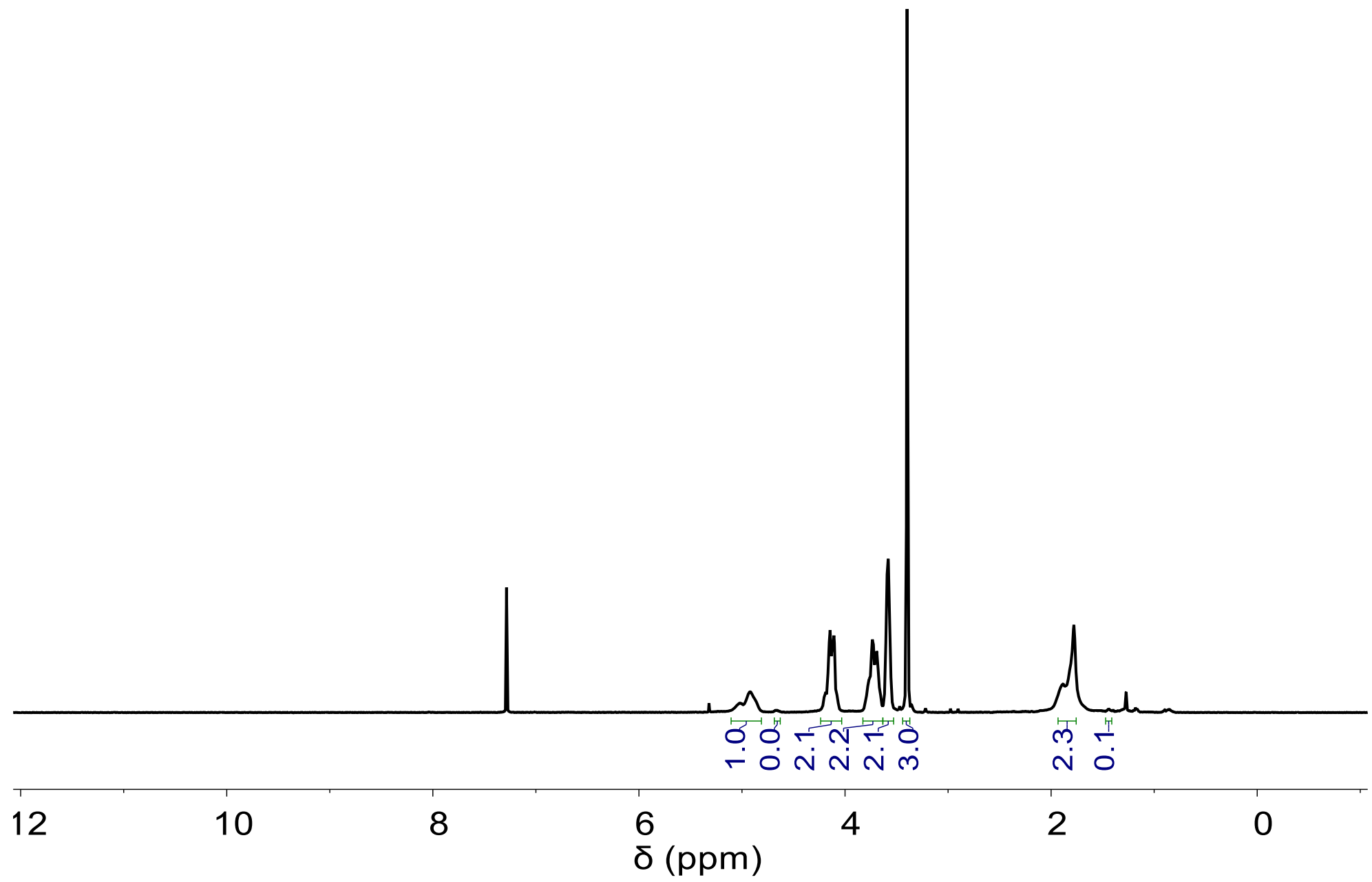


**Figure S16:** $^1$H-NMR spectrum (400MHz, $CDCl_3$) of PMEGVE-CTA. δ 5.20 – 4.76 (m, 1H, $CH_2$-C*H*- backbone), 4.68 – 4.66 (m, 2H, O-C$H_2$- end-group), 4.24 – 4.06 (m, 2H, O-C$H_2$- side chain), 3.82 – 3.65 (m, 2H, O-C$H_2$- side chain), 3.64 – 3.53 (m, 2H, COO-C$H_2$-O side chain), 3.40 (s, 3H, -C$H_3$ side chain), 1.99 – 1.70 (m, 2H, CH-C$H_2$- backbone), 1.49 – 1.42 (m, 3H, $CH_2$-C$H_3$ end-group). $DP_{exp}$ = 55.3, $M_{n,NMR}$ = 8.9 kDa.

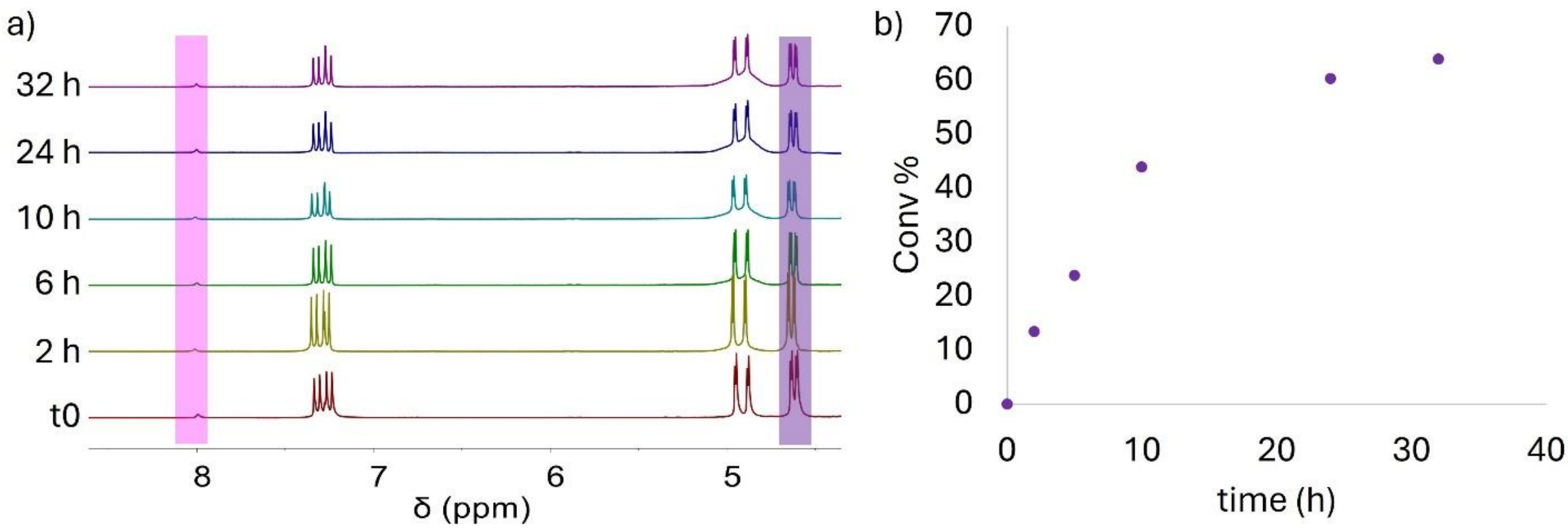


**Figure S17: a)** Kinetics of RAFT/MADIX polymerization of MEGVE evaluated by $^1$H-NMR (200MHz, $CDCl_3$), the signal of DMF (used as reference) and MEGVE (used for conversion calculation) are highlighted. **b)** Conversion *vs* time during RAFT/MADIX polymerization of MEGVE.

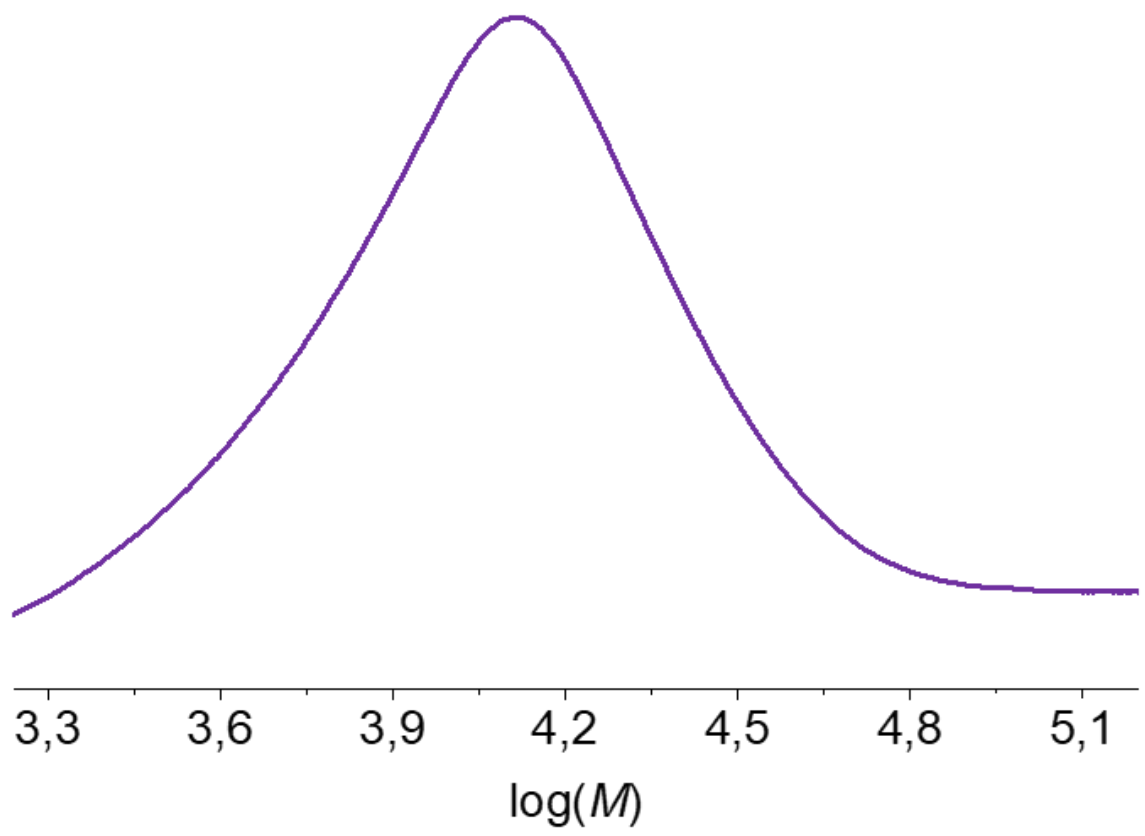


**Figure S18:** SEC trace for PMEGVE-CTA (eluent DMF with 1 g $L^{-1}$ LiBr). $M_{n,SEC}$ = 9.1 kDa, $Đ$ = 1.50

### 3.1.2 PMEGA-CTA

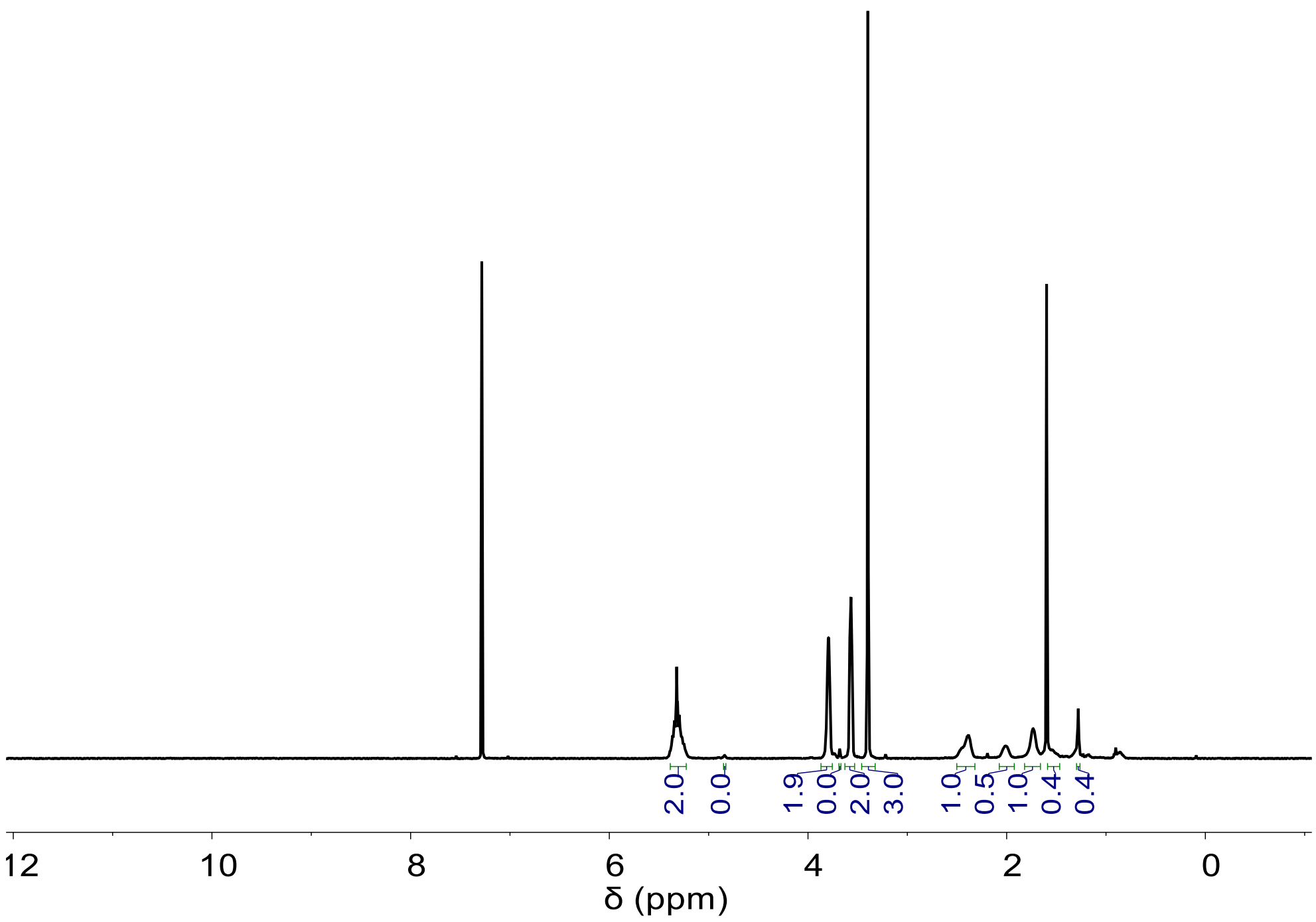


**Figure S19:** $^1$H-NMR spectrum (400MHz, $CDCl_3$) of PMEGA-CTA. δ 5.39 – 5.23 (m, 2H, O-C$H_2$-O side chain), 4.87 – 4.80 (m, 1H, -C*H*- end-goup), 3.87 – 3.76 (m, 2H, O-C$H_2$- side chain), 3.69 – 3.67 (m, 3H, O-C$H_3$ end-goup), 3.63 – 3.53 (m, 2H, O-C$H_2$- side chain), 3.40 (s, 3H, O-C$H_3$ side chain), 2.50 – 2.32 (m, 1H, CH-C$H_2$- backbone), 2.07 – 1.96 (m, 0.5H, $CH_2$-C*H*- backbone), 1.76 – 1.71 (m, 1H, CH-C$H_2$- backbone), 1.63 – 1.49 (m, 0.5H, $CH_2$-C*H*- backbone), 1.28 (s, 18H, -C$H_2$- end-goup). $DP_{exp}$ = 56.3 $M_{n,NMR}$ = 9.0 kDa

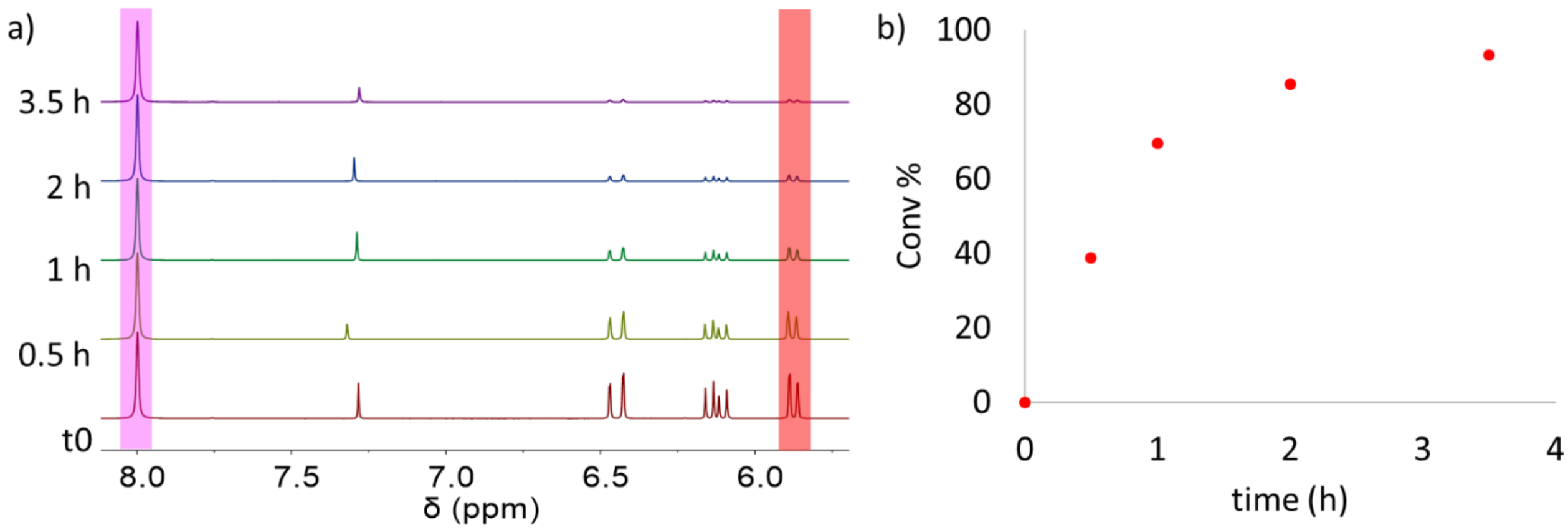


**Figure S20: a)** Kinetics of RAFT polymerization of MEGA monitored by $^{1}$H-NMR (400MHz, $CDCl_3$), signals from DMF (used as reference) and MEGA (used for conversion calculation) are highlighted. **b)** Conversion *vs* time during RAFT polymerization of MEGA.

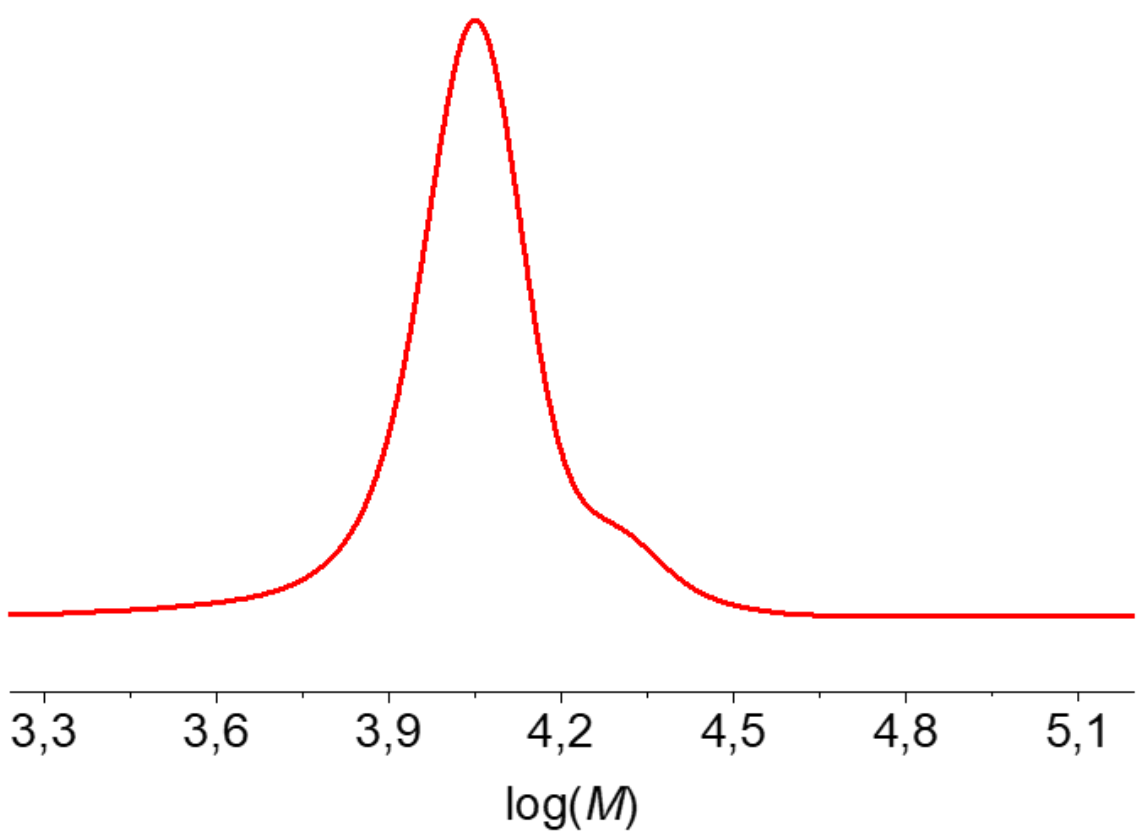


**Figure S21:** SEC trace of PMEGA-CTA (DMF with 1 g/L LiBr). $M_{n,SEC}$ = 10.9 kDa, $Đ$ = 1.09.

### 3.1.3 PDEGA-CTA

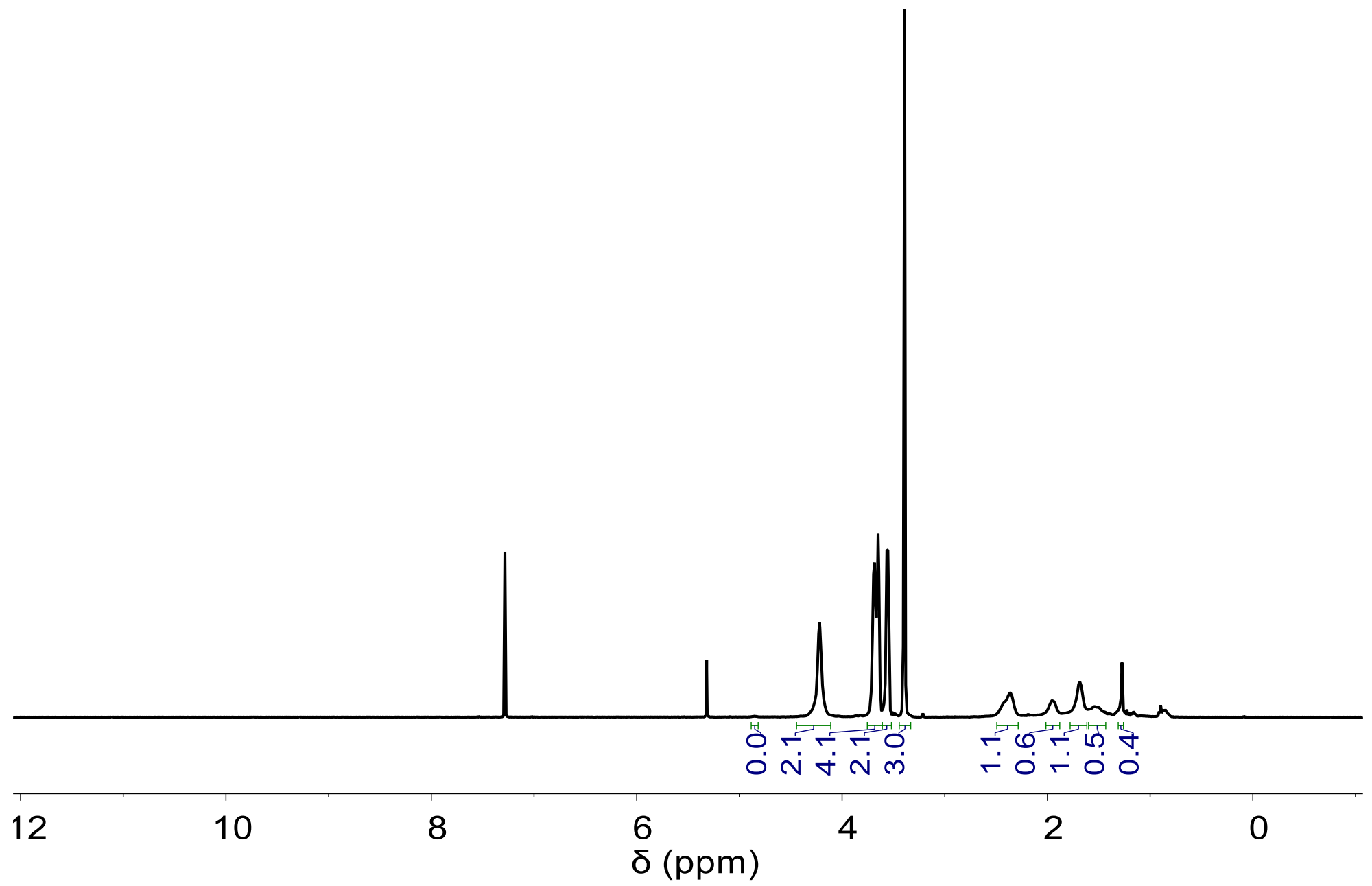


**Figure S22:** $^{1}$H-NMR spectrum (400MHz, $CDCl_3$) of PDEGA-CTA. δ 4.89 – 4.82 (m, 1H, -C*H*- end-goup), 4.44 – 4.11 (m, 2H, O-C$H_2$- side chain), 3.75 – 3.61 (m, 4H, O-C$H_2$- side chain), 3.61 – 3.52 (m, 2H, O-C$H_2$- side chain), 3.39 (s, 3H, O-C$H_3$ side chain), 2.49 – 2.28 (m, 1H, CH-C$H_2$- backbone), 2.01 – 1.88 (m, 0.5H, $CH_2$-C*H*- backbone), 1.77 – 1.62 (m, 1H, CH-C$H_2$- backbone), 1.60 – 1.42 (m, 0.5H, $CH_2$-C*H*- backbone), 1.27 (s, 18H, -C$H_2$- end-goup). $DP_{exp}$ = 51.0, $M_{n,NMR}$ = 8.9 kDa.

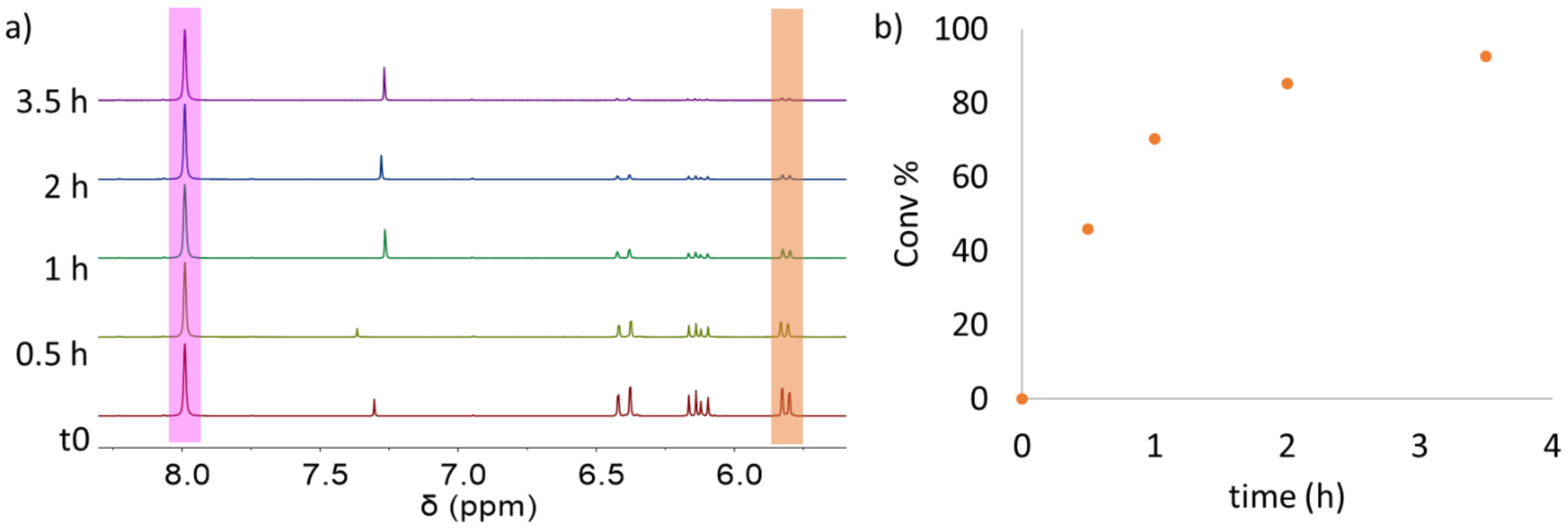


**Figure S23: a)** Kinetics of RAFT polymerization of DEGA monitored by $^{1}$H-NMR (400MHz, $CDCl_3$). Signals from DMF (used as reference) and DEGA are highlighted. **b)** Conversion *vs* time recorded during RAFT polymerization of DEGA.

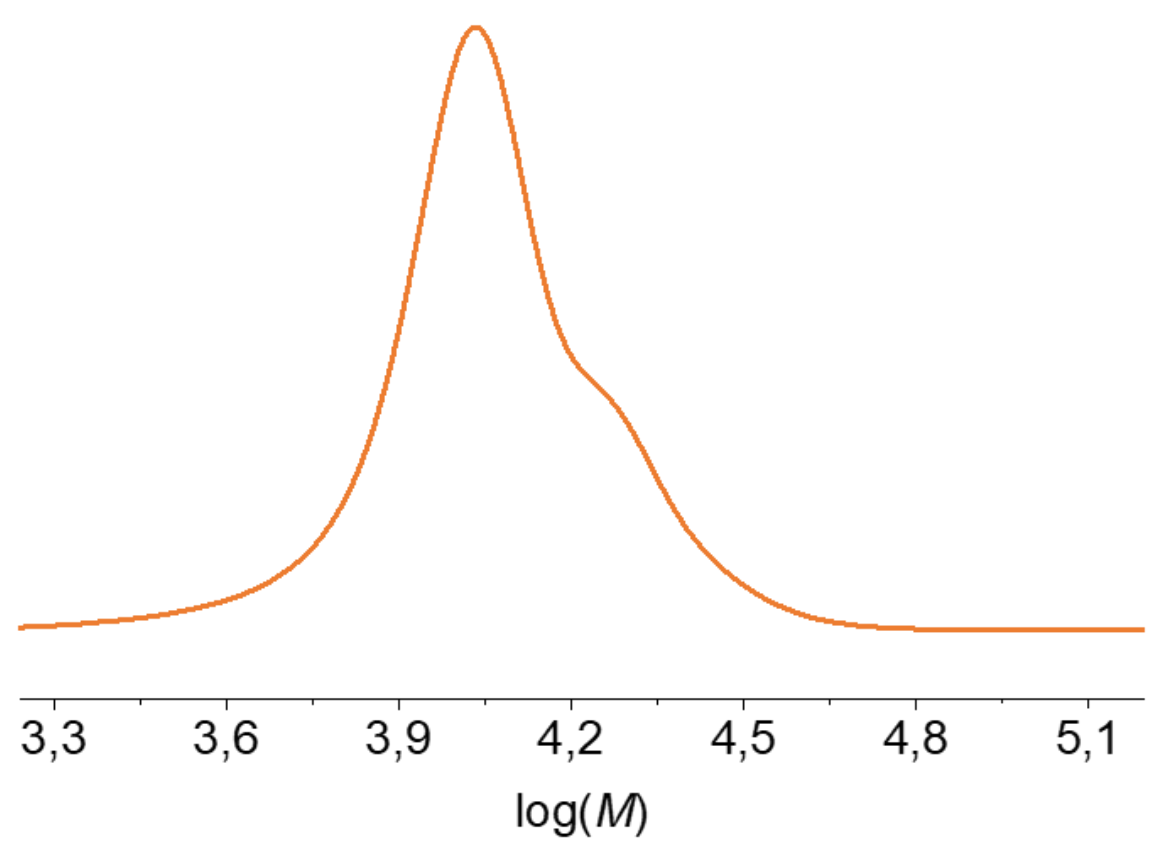


**Figure S24:** SEC trace of PDEGA-CTA (DMF with 1 g/L LiBr). $M_{n,SEC}$ = 10.5 kDa, $Đ$ = 1.19

## 3.1.4 PDEGVE-CTA

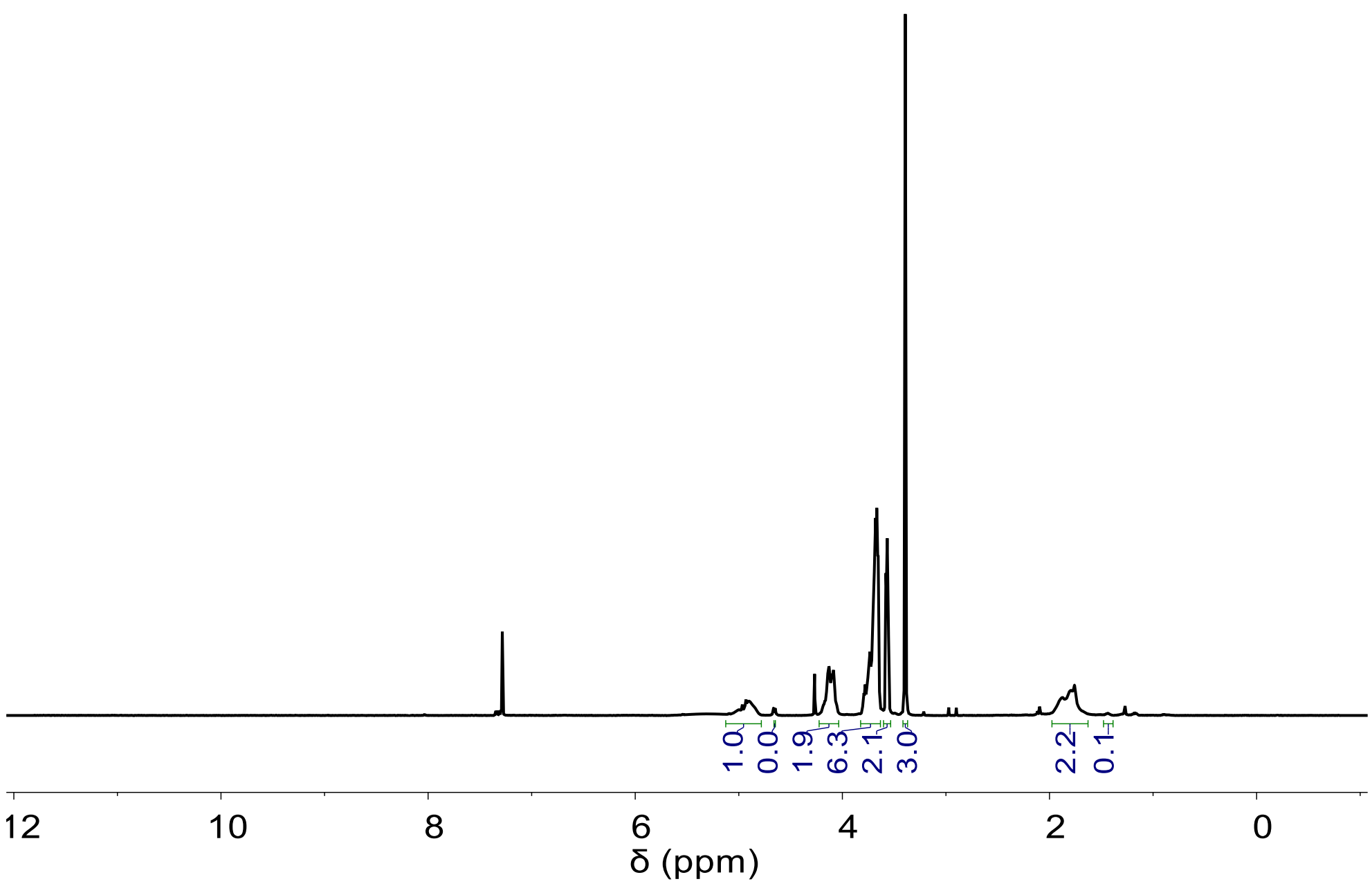


**Figure S25:** $^{1}$H-NMR spectrum (400MHz, $CDCl_3$) of PDEGVE-CTA. δ 5.13 – 4.76 (m, 1H, $CH_2$-C*H*- backbone), 4.69 – 4.62 (m, 2H, O-C$H_2$- end-group), 4.24 – 4.02 (m, 2H, O-C$H_2$- side chain), 3.82 – 3.63 (m, 4H, O-C$H_2$- side chain and 2H, COO-C$H_2$-O side chain ), 3.57 (t, $J$ = 4.8 Hz, 2H, O-C$H_2$- side chain), 3.39 (s, 3H, -C$H_3$ side chain), 1.98 – 1.61 (m, 2H, CH-C$H_2$- backbone), 1.48 – 1.39 (m, 3H, $CH_2$-C$H_3$ end-group). $DP_{exp}$ = 59.6, $M_{n,NMR}$ = 12.2 kDa.

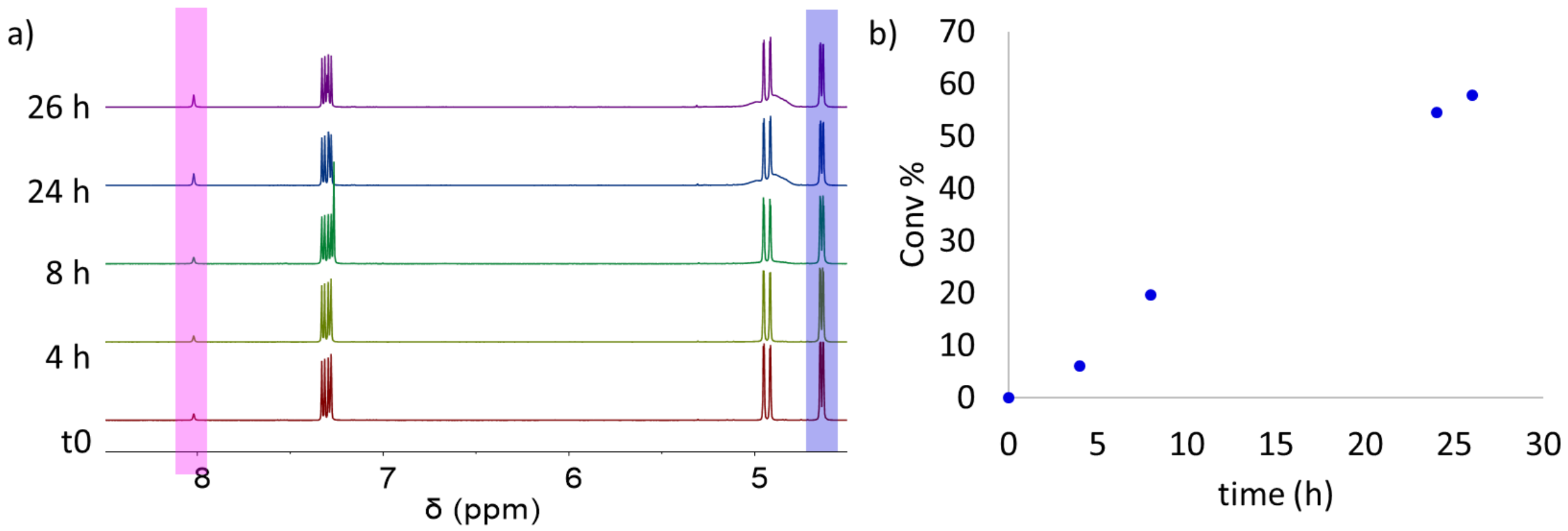


**Figure S26: a)** Kinetics of RAFT/MADIX polymerization of DEGVE monitored by $^{1}$H-NMR (400MHz, $CDCl_3$), signals from DMF (used as reference) and DEGA (used for conversion calculation) are highlighted. **b)** Conversion *vs* time recorded during RAFT/MADIX polymerization of DEGA.

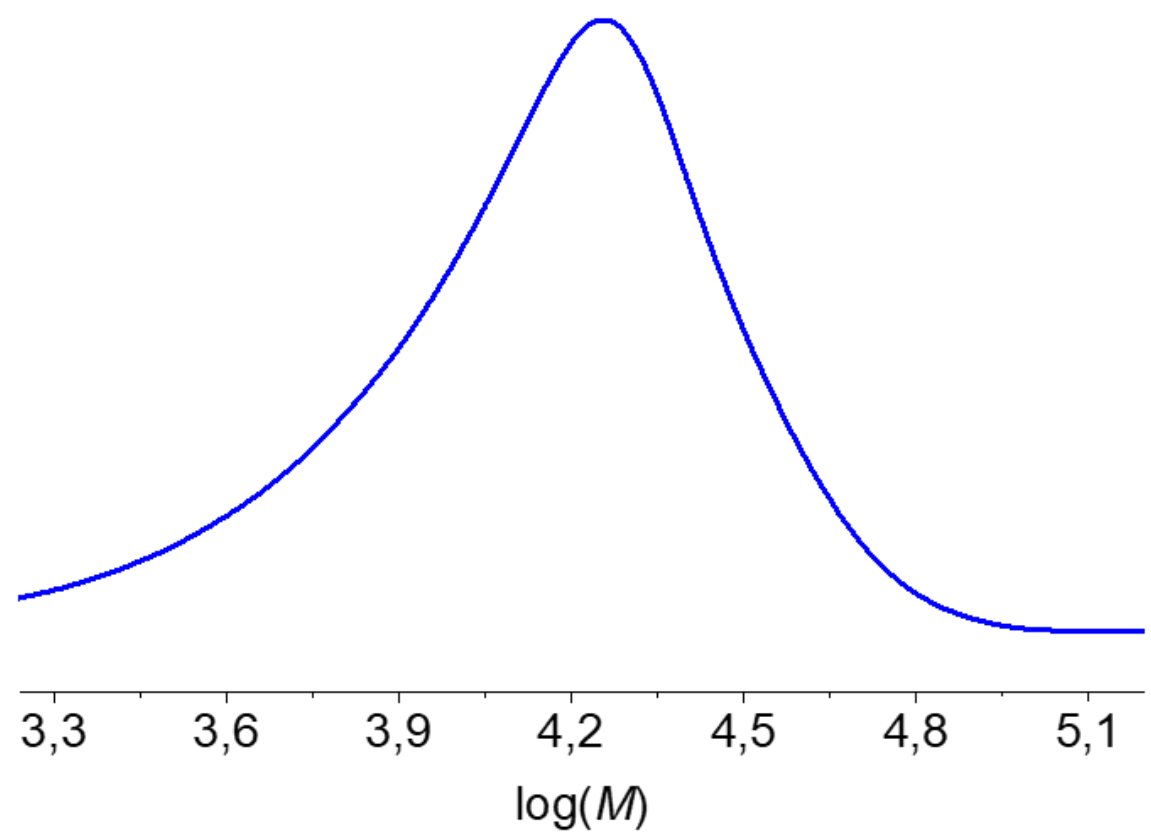


**Figure S27:** SEC trace of PDEGVE-CTA (DMF with 1 g/L LiBr, 5 mg/mL, PMMA calibration). $M_{n,SEC}$ = 11.3 kDa, $Đ$ = 1.51.

## 3.2 Post-polymerization modification

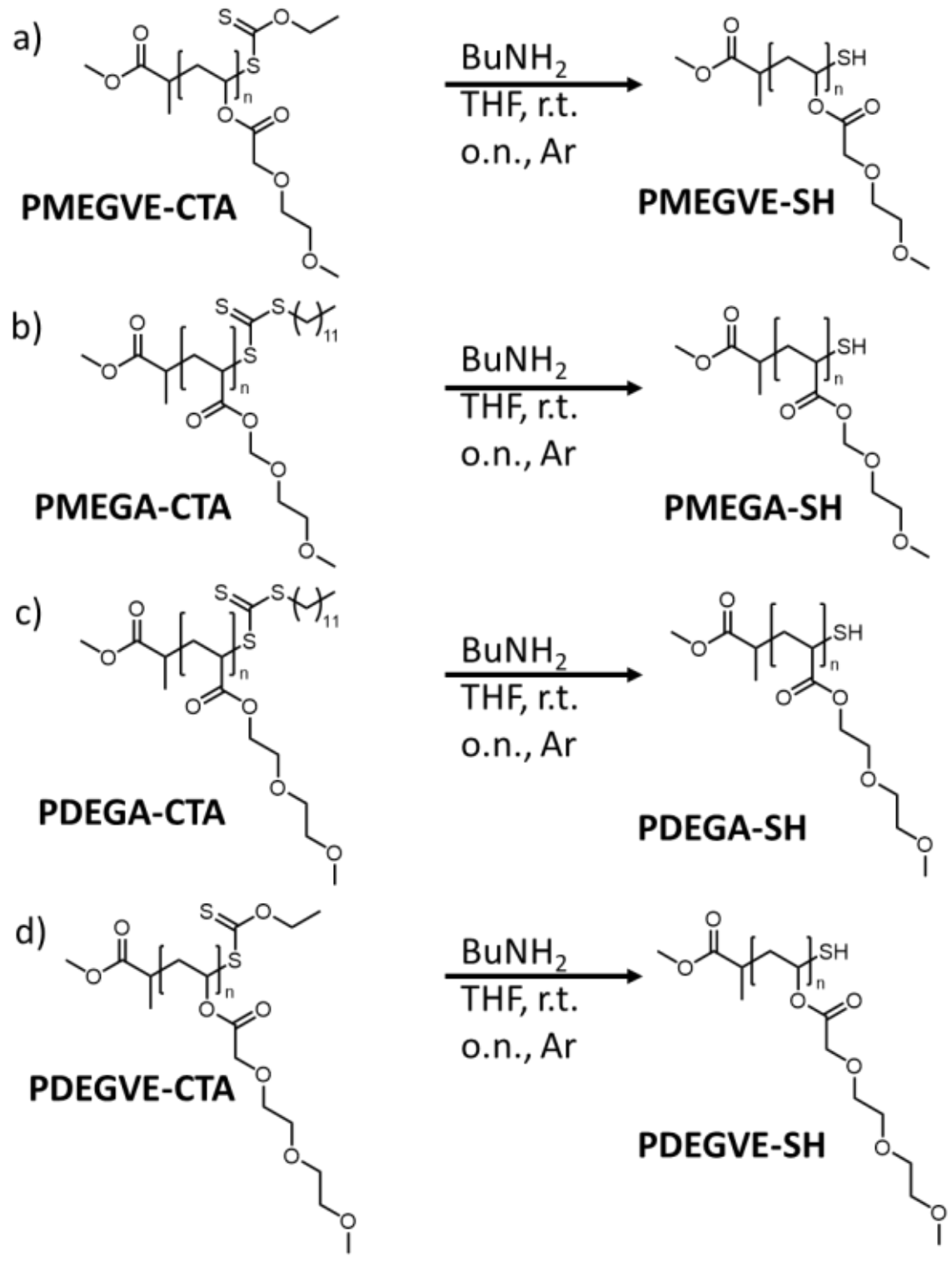


**Figure S28:** Synthetic scheme for end-group removal for the preparation of **a)** PMEGVE-SH, **b)** PDEGVE-SH, **c)** PMEGA-SH and **d)** PDEGA-SH.

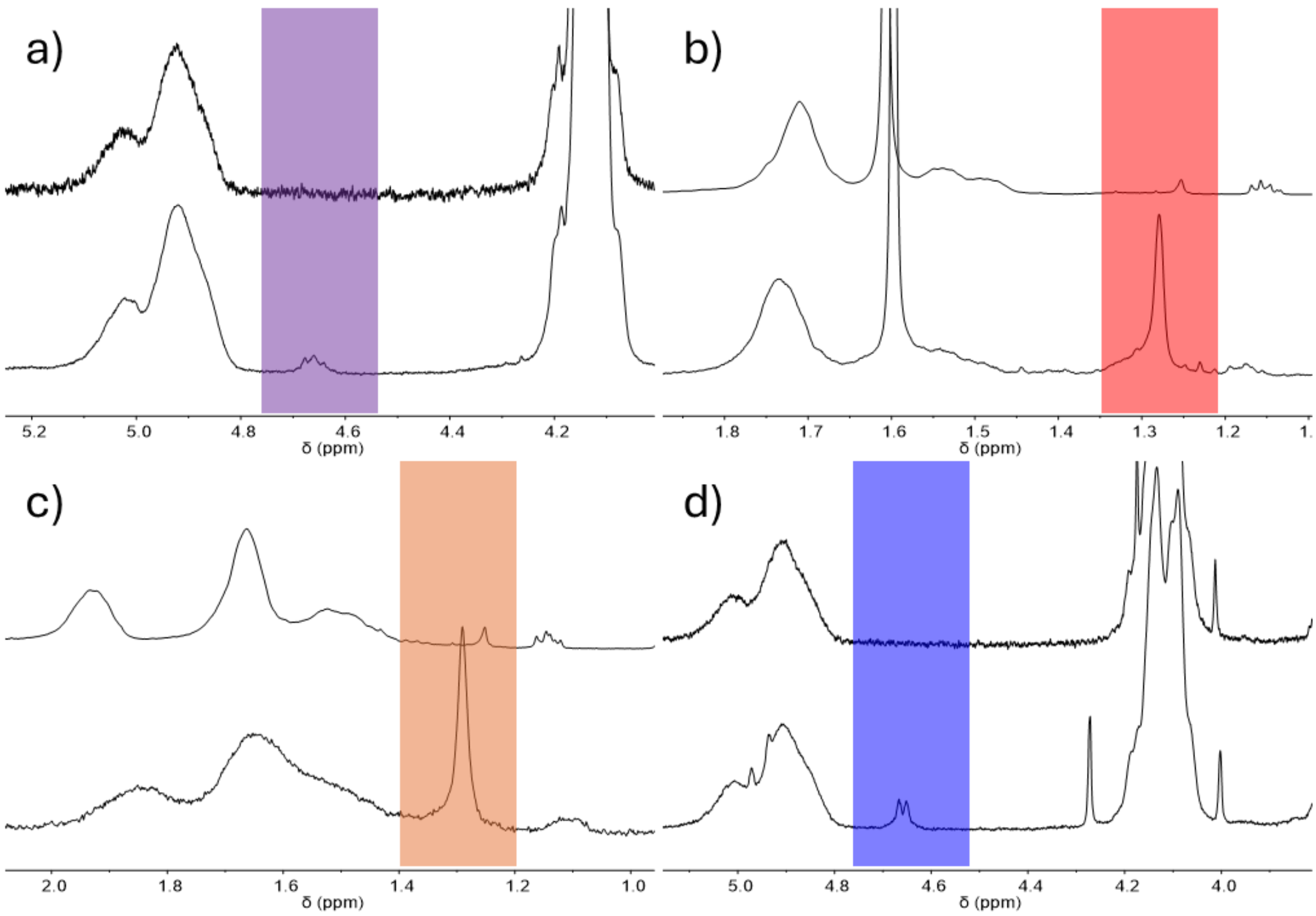


**Figure S29:** $^1$H-NMR zoomed spectrum of one of the CTAs signals before (bottom) and after (top) the reaction with $BuNH_2$, of **a)** PMEGVE, **b**) PMEGA, **c)** PDEGA and **d)** PDEGVE.

### 3.2.1 PMEGVE-SH

**LCST:** ($H_2O$, 5 mg/mL, 1 °C/min, 700 nm) $T_{Heating}$ = 65.2 °C; $T_{cooling}$ = 62.1 °C.

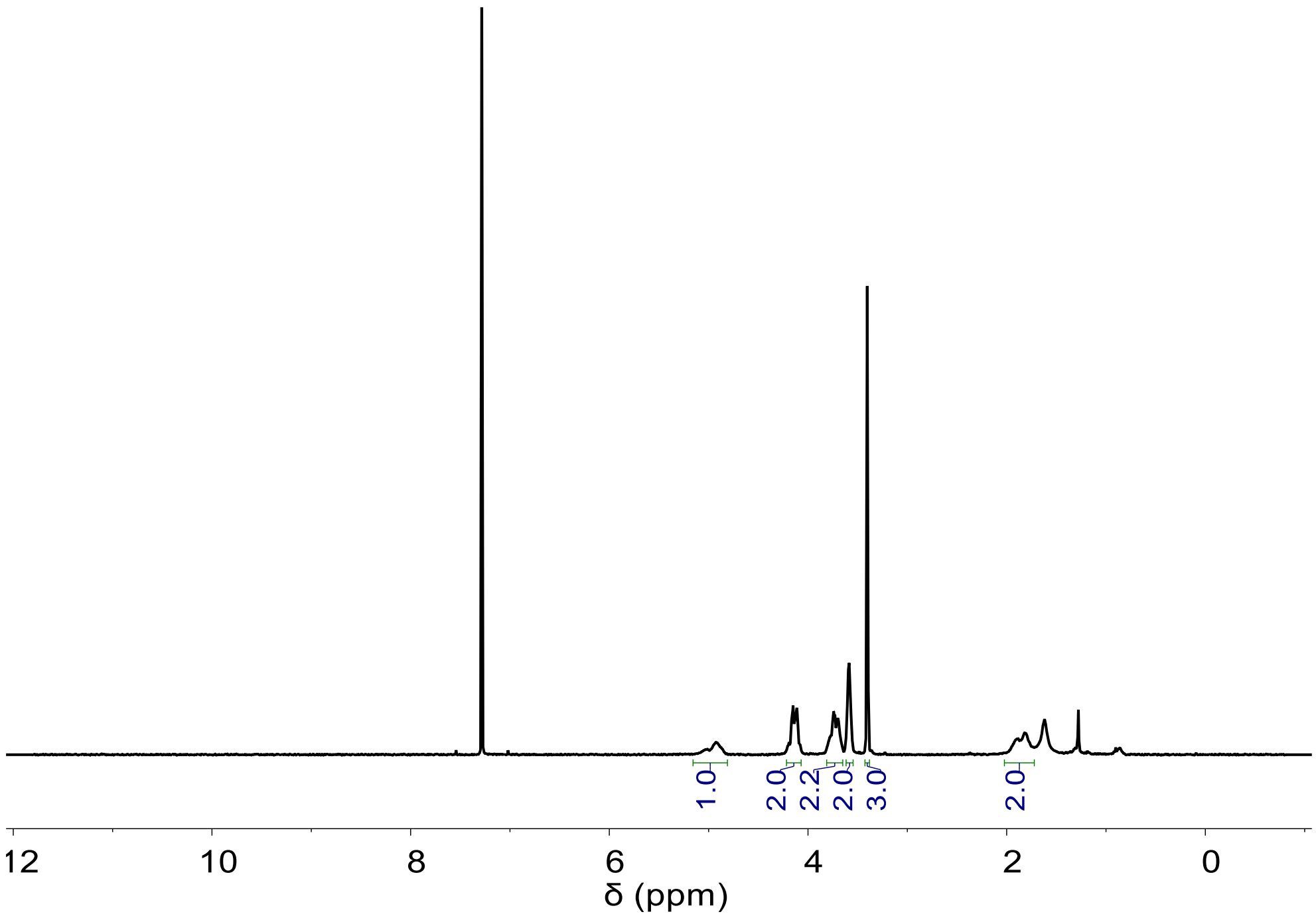


**Figure S30:** $^1$H-NMR spectrum (400MHz, $CDCl_3$) of PMEGVE-SH. δ 5.16 – 4.81 (m, 1H, $CH_2$-C*H*- backbone), 4.24 – 4.06 (m, 2H, O-C$H_2$- side chain), 3.81 – 3.65 (m, 2H, O-C$H_2$- side chain), 3.62 – 3.54 (m, 2H, COO-C$H_2$-O side chain), 3.40 (s, 3H, -C$H_3$ side chain), 2.02 – 1.72 (m, 2H, CH-C$H_2$- backbone).

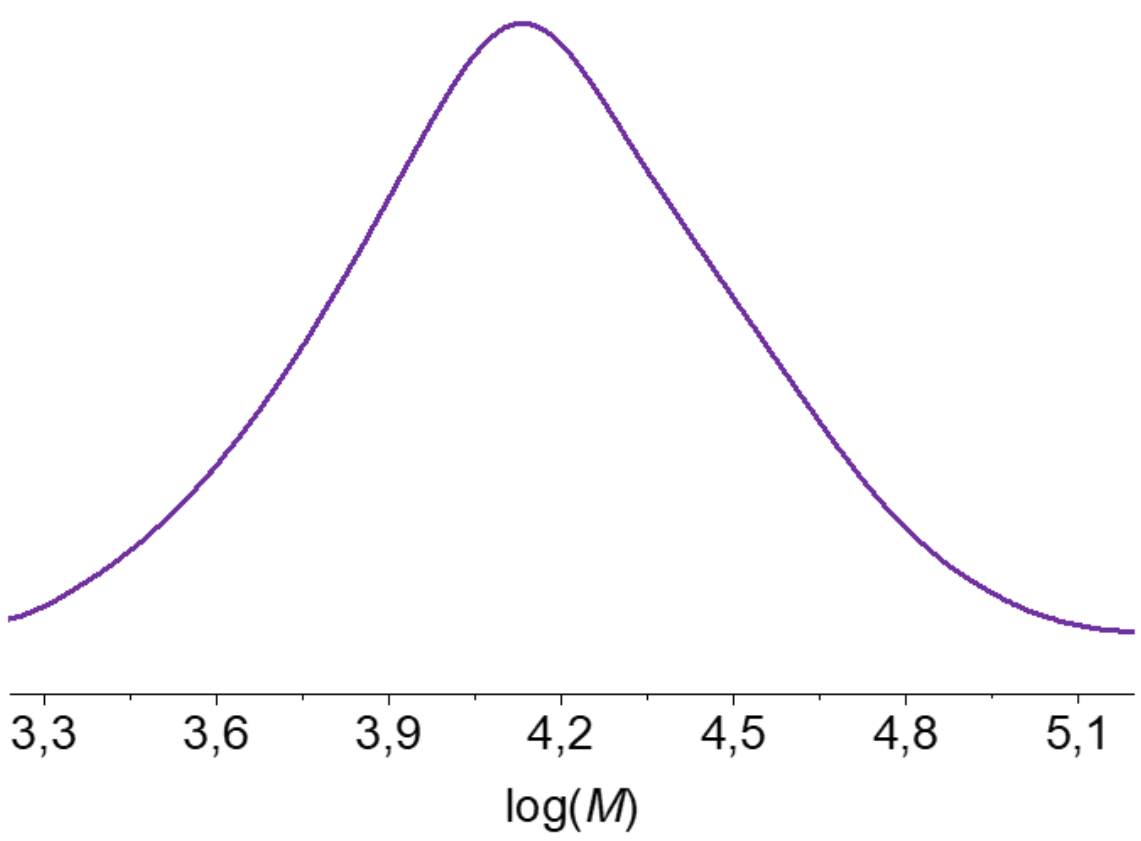


**Figure S31:** SEC trace of PMEGVE-SH (DMF with 1 g/L LiBr). $M_{n,SEC}$ = 10.3 kDa, $Đ$ = 1.67.

## 3.2.2 PMEGA-SH

**LCST:** ($H_2O$, 5 mg/mL, 1 °C/min, 700 nm) $T_{Heating}$ = 12.3 °C; $T_{cooling}$ = 9.1 °C.

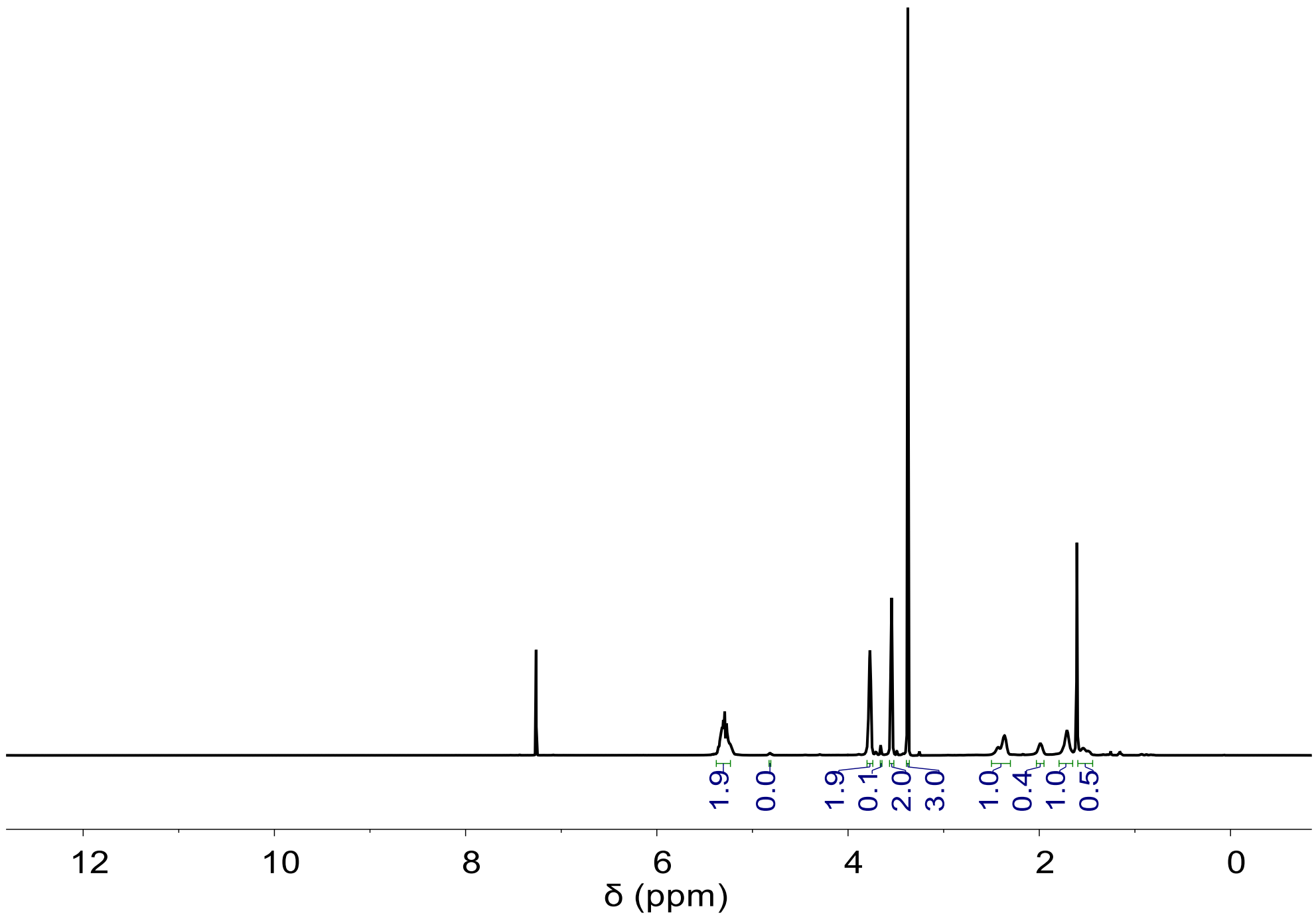


**Figure S32:** $^{1}$H-NMR spectrum (600MHz, $CDCl_3$) of PMEGA-SH. δ 5.38 – 5.23 (m, 2H, O-C*H₂*-O side chain), 4.87 – 4.78 (m, 1H, -C*H*- end-goup), 3.77 (t, *J* = 6.1 Hz, 2H, O-$CH_2$- side chain), 3.67 – 3.65 (m, 3H, O-$CH_3$ end-group), 3.54 (t, *J* = 6.1 Hz, 2H, O-$CH_2$- side chain), 3.37 (s, 3H, O-$CH_3$ side chain), 2.50 – 2.30 (m, 1H, CH-$CH_2$- backbone), 2.03 – 1.95 (m, 0.5H, $CH_2$-C*H*- backbone), 1.79 – 1.65 (m, 1H, CH-$CH_2$- backbone), 1.60 – 1.44 (m, 1H, $CH_2$-C*H*- backbone).

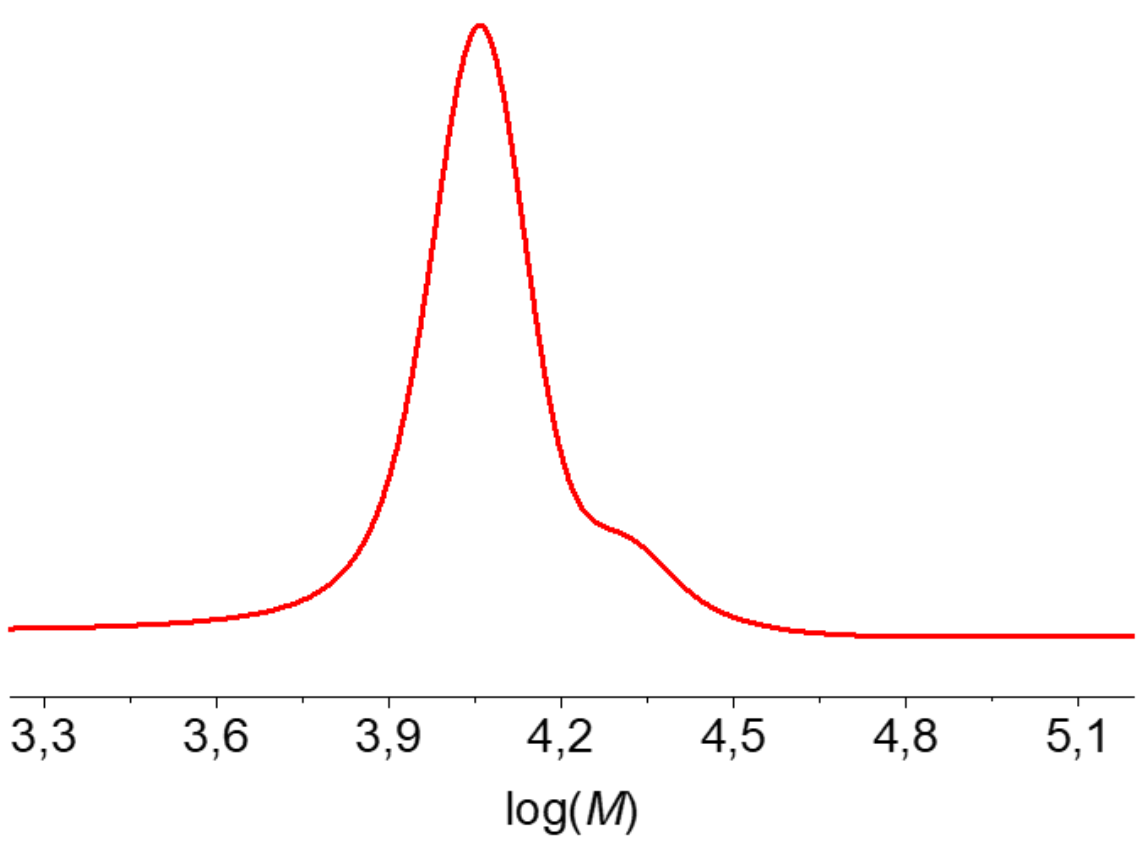


**Figure S33:** SEC trace of PMEGA-SH (DMF with 1 g/L LiBr). $M_{n,SEC}$ = 11.1 kDa, $Đ$ = 1.11.

### 3.2.3 PDEGA-SH

**LCST:** ($H_2O$, 5 mg/mL, 1 °C/min, 700 nm) $T_{Heating}$ = 47.3 °C; $T_{cooling}$ = 43.6 °C.

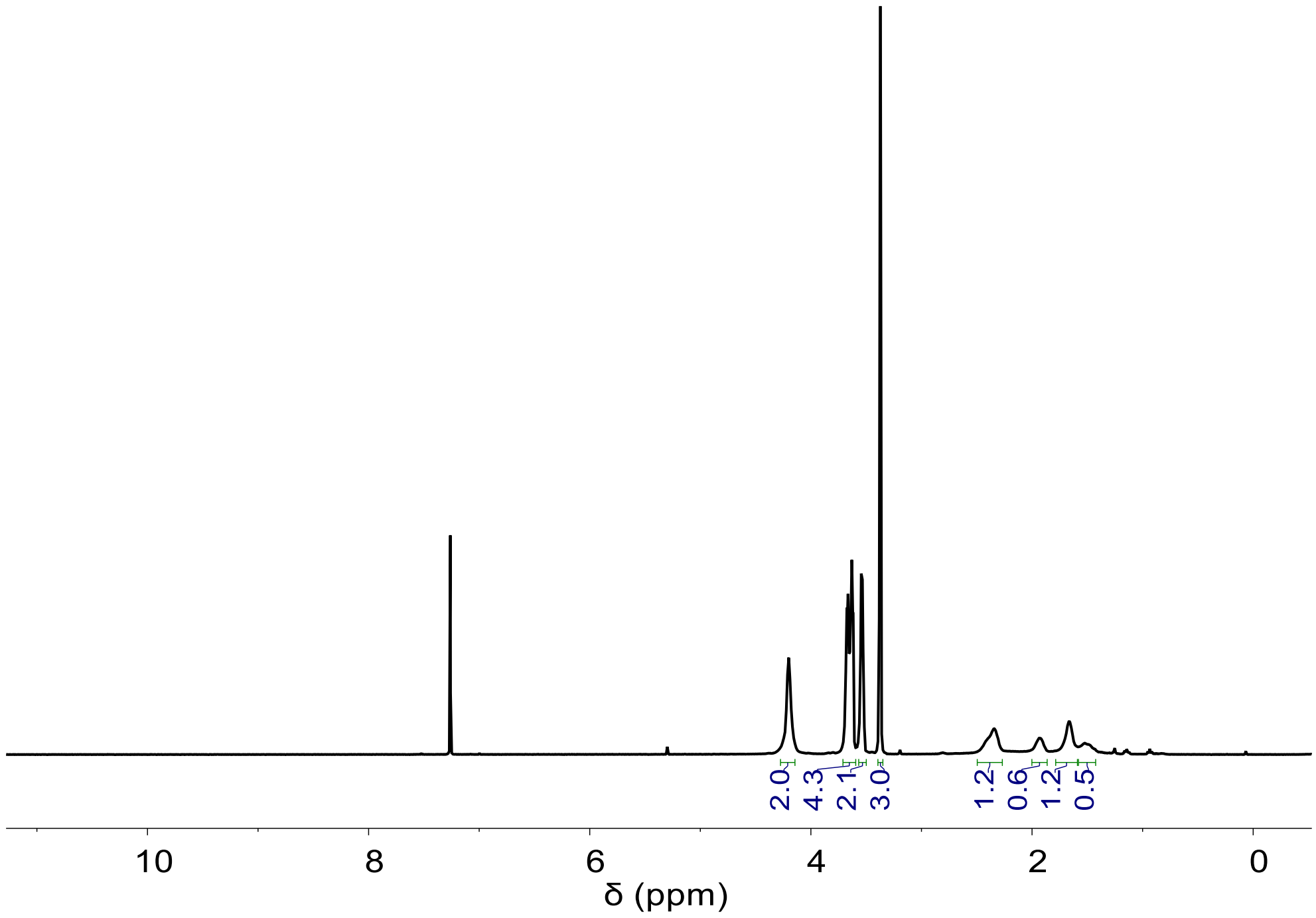


**Figure S34:** $^{1}$H-NMR spectrum (400MHz, $CDCl_3$) of PDEGA-SH. δ 4.27 – 4.14 (m, 2H, O-C$H_2$- side chain), 3.71 – 3.59 (m, 4H, O-C$H_2$- side chain), 3.57 – 3.50 (m, 2H, O-C$H_2$- side chain), 3.37 (s, 3H, O-$CH_3$ side chain), 2.50 – 2.27 (m, 1H, CH-C$H_2$- backbone), 2.00 – 1.86 (m, 0.5H, $CH_2$-C*H*- backbone), 1.79 – 1.59 (m, 1H, CH-C$H_2$- backbone), 1.58 – 1.42 (m, 0.5H, $CH_2$-C*H*- backbone).

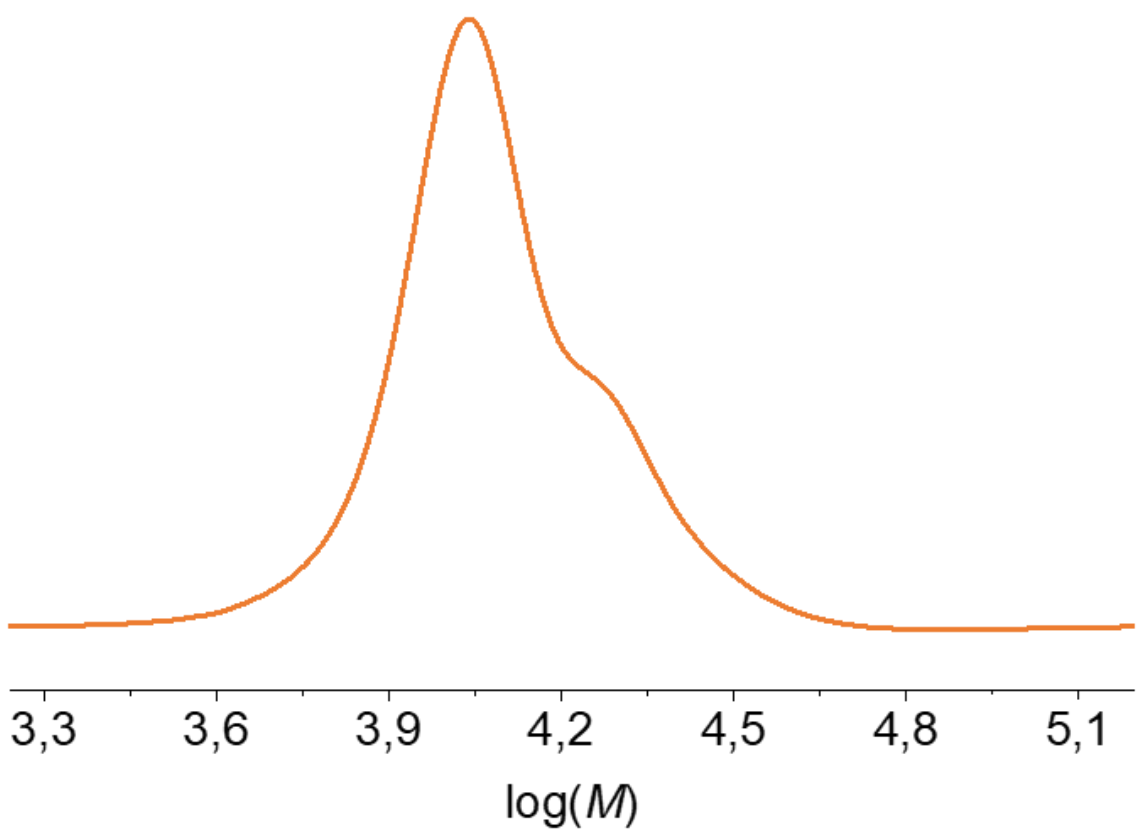


**Figure S35:** SEC trace of PDEGA-SH (DMF with 1 g/L LiBr). $M_{n,SEC}$ = 10.7 kDa, $Đ$ = 1.17.

## 3.2.4 PDEGVE-SH

**LCST:** ($H_2O$, 5 mg/mL, 1 °C/min, 700 nm) $T_{Heating}$ = 87.5 °C; $T_{cooling}$ = 84.0 °C.

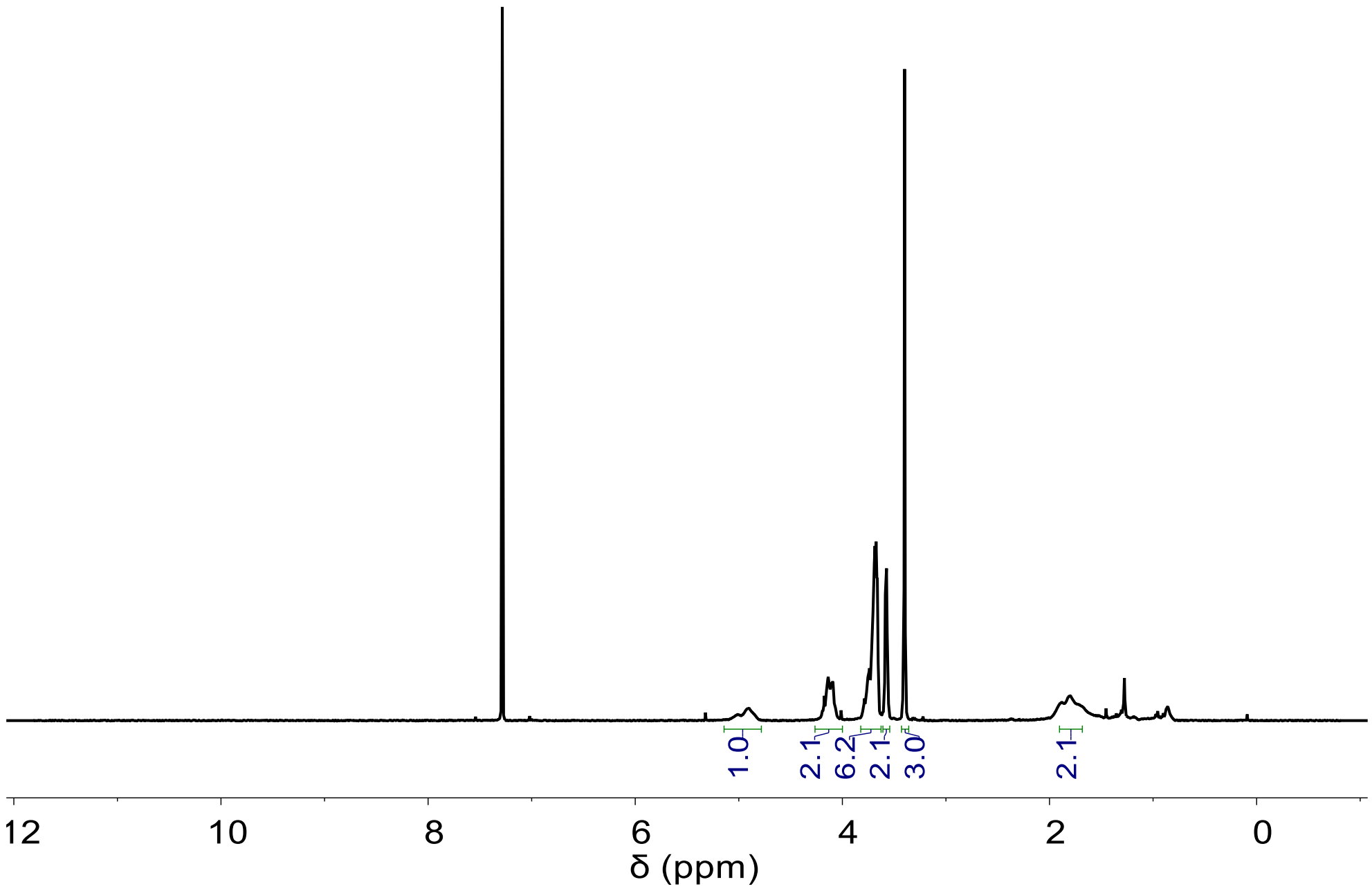


**Figure S36:** $^1$H-NMR spectrum (400MHz, $CDCl_3$) of PDEGVE-SH. δ 5.01 – 4.90 (m, 1H, $CH_2$-C*H*- backbone), 4.17 – 4.01 (m, 2H, O-C$H_2$- side chain), 3.79 – 3.66 (m, 4H, O-C$H_2$- side chain and 2H, COO-C$H_2$-O side chain), 3.59 – 3.56 (m, 2H, O-C$H_2$- side chain), 3.40 (s, 3H, -C$H_3$ side chain), 1.88 – 1.71 (m, 2H, CH-C$H_2$- backbone).

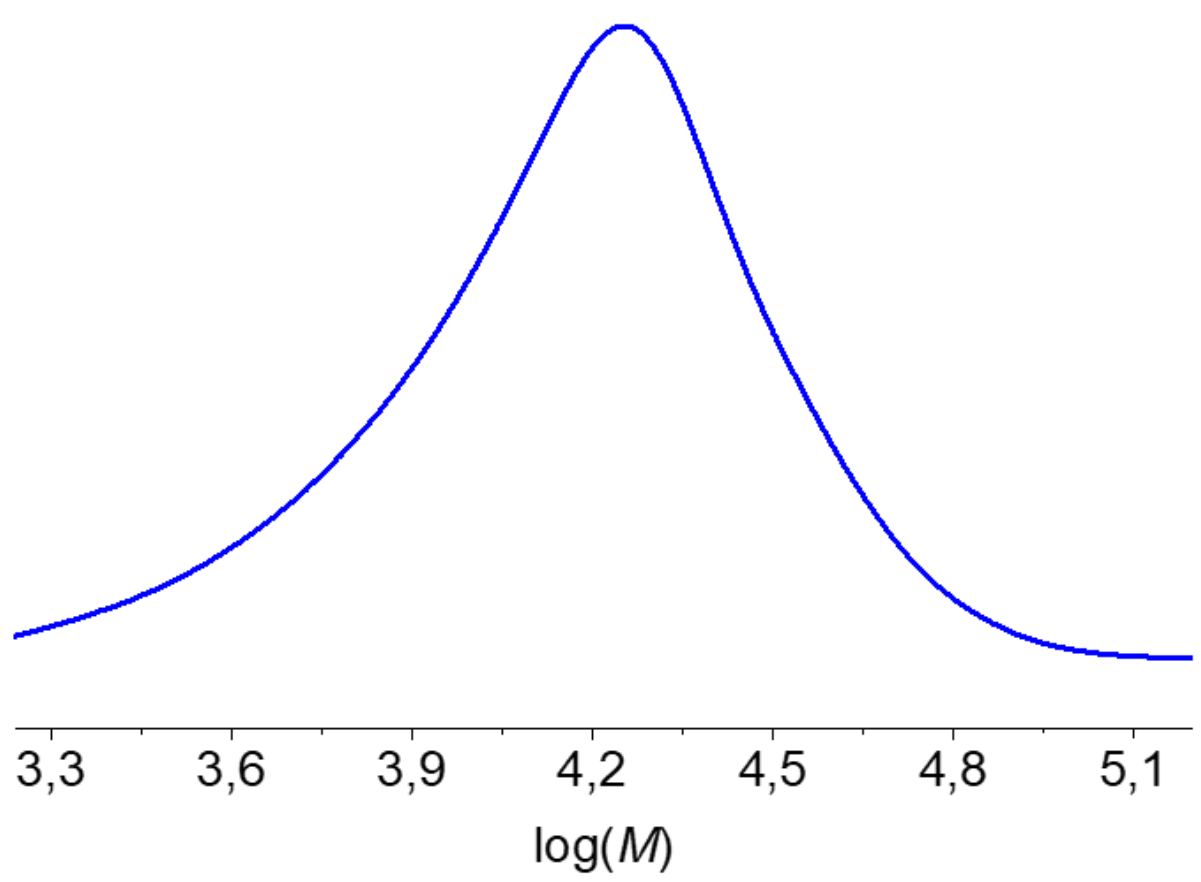


**Figure S37:** SEC trace of PDEGVE-SH (DMF with 1 g/L LiBr). $M_{n,SEC}$ = 11.5 kDa, *Đ* = 1.56.

## 3.3 QCMD Sensograms

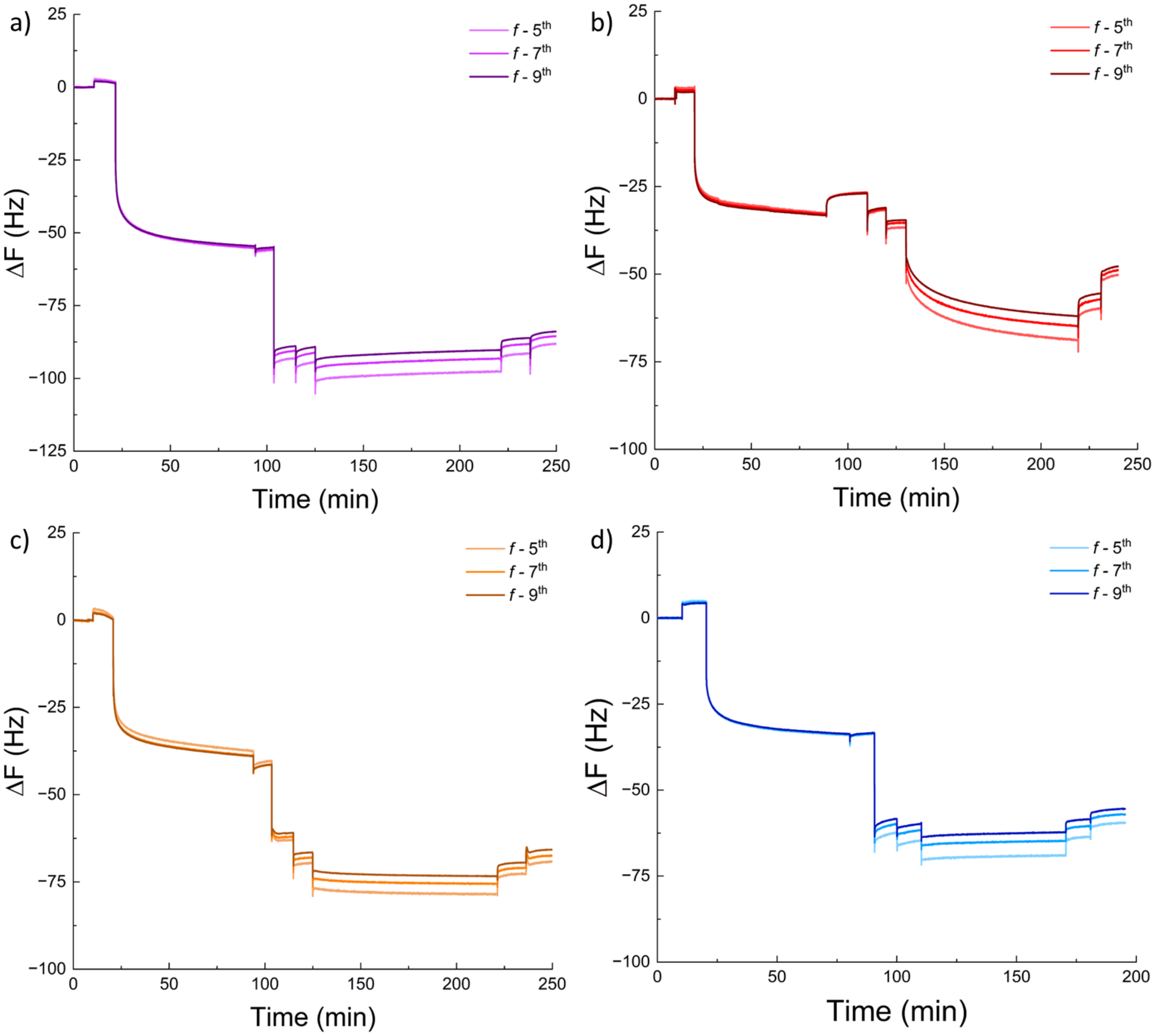


**Figure S38:** QCMD sensograms reporting the variation of ΔF during the assembly of different brushes on Au-coated sensors and upon interaction with protein-rich media. **a)** PMEGVE, **b**) PMEGA, **c)** PDEGA and **d)** PDEGVE.
